# Neutralizing Antibody Titers in Hospitalized Patients with Acute Puumala Orthohantavirus Infection Do Not Associate with Disease Severity

**DOI:** 10.3390/v14050901

**Published:** 2022-04-26

**Authors:** Rommel Iheozor-Ejiofor, Katariina Vapalahti, Tarja Sironen, Lev Levanov, Jussi Hepojoki, Åke Lundkvist, Satu Mäkelä, Antti Vaheri, Jukka Mustonen, Alexander Plyusnin, Tomas M. Strandin, Olli Vapalahti

**Affiliations:** 1 Department of Virology, Medicum, University of Helsinki, 00014 Helsinki, Finland; tarja.sironen@helsinki.fi (T.S.); lev.levanov@helsinki.fi (L.L.); jussi.hepojoki@helsinki.fi (J.H.); antti.vaheri@helsinki.fi (A.V.); alexander.plyusnin@helsinki.fi (A.P.); tomas.strandin@helsinki.fi (T.M.S.); olli.vapalahti@helsinki.fi (O.V.); 2 Department of Veterinary Biosciences, University of Helsinki, 00014 Helsinki, Finland; katariina.vapalahti@helsinki.fi; 3 Diagnostic Center, Virology and Immunology, HUSLAB, Helsinki University Hospital, 00029 Helsinki, Finland; 4 Vetsuisse Faculty, Institute of Veterinary Pathology, University of Zürich, CH-8057 Zürich, Switzerland; 5 Department of Medical Biochemistry and Microbiology, Zoonosis Science Center, Uppsala University, SE-751 05 Uppsala, Sweden; ake.lundkvist@imbim.uu.se; 6 Department of Internal Medicine, Tampere University Hospital, 33520 Tampere, Finland; satu.makela@pshp.fi (S.M.); jukka.mustonen@tuni.fi (J.M.); 7 Faculty of Medicine and Health Technology, Tampere University, 33014 Tampere, Finland

**Keywords:** nephropathia epidemica, AKI, pFRNT, disease severity, neutralizing antibody

## Abstract

Nephropathia epidemica (NE), a mild form of haemorrhagic fever with renal syndrome (HFRS), is an acute febrile illness caused by Puumala orthohantavirus (PUUV). NE manifests typically with acute kidney injury (AKI), with a case fatality rate of about 0.1%. The treatment and management of hantavirus infections are mainly supportive, although neutralizing monoclonal antibodies and immune sera therapeutics are under investigation. In order to assess the potential use of antibody therapeutics in NE, we sought to determine the relationship between circulating PUUV neutralizing antibodies, PUUV nucleocapsid protein (N) IgG antibodies, and viral loads with markers of disease severity. The study included serum samples of extensively characterized patient cohorts (*n* = 116) from Tampere University Hospital, Finland. The results showed that upon hospitalization, most patients already had considerable neutralizing and anti-PUUV-N IgG antibody levels. However, contrary to expectations, neutralizing antibody titers from the first day of hospitalization did not appear to protect from AKI or correlate with more favorable disease outcomes. This indicates that further studies are needed to investigate the applicability of neutralizing antibodies as a therapy for hospitalized NE patients.

## 1. Introduction

Puumala hantavirus (PUUV) is the causative agent of a mild form of haemorrhagic fever with renal syndrome (HFRS), also known as nephropathia epidemica (NE) [1,2]. PUUV is an enveloped virus with a trisegmented negative strand RNA genome belonging to the order *Bunyavirales*, family *Hantaviridae*, genus *Orthohantavirus*, and is carried by its rodent host, the bank vole (*Myodes glareolus*) [3,4], which inhabits the forested regions of Europe. PUUV infection in the bank vole is subclinical and persistent, with a short-lived viremia and prolonged virus shedding in saliva and faces [5]. Humans are incidental hosts of PUUV, and the infection presents as NE, with varying severity [6,7].

Between 2014 and 2018, an average of about 3000 (ranging from 1826 to 4249) NE cases were reported annually in the European Union [8], with the case fatality rate being about 0.1%. Finland, Sweden, and Germany comprised 83%, and Finland alone 53%, of all cases within the same period [8]. The pathogenesis, which is likely connected to the PUUV infection of microvascular endothelial cells, has been discussed extensively previously [9,10,11]. Clinical symptoms of varying severity usually develop after a 2–4-week incubation period. Symptoms include a rapid onset of fever, headache, gastrointestinal symptoms such as abdominal pains and nausea, dizziness, and ophthalmological manifestations (including myopia in about third of the patients). Thrombocytopenia, leukocytosis, proteinuria, hematuria, elevated serum C-reactive protein, and creatinine levels are typical laboratory findings. Hypotension, hemorrhages, pethecchiae, disseminated intravascular coagulation (DIC), cardiac symptoms, or hypophyseal haemorrhage, which can be fatal [11], may occur in the more severe cases. Renal involvement becomes apparent one week after the onset of symptoms, first with oliguria or anuria, elevated serum creatinine levels, proteinuria, and hematuria, which are then followed by polyuria in the second week after the onset of infection.

We recently developed pseudotype viruses with PUUV glycoproteins Gn and Gc expressed on the surface of a replication-defective vesicular stomatitis virus (VSV) particle [12]. These pseudotype viruses produce enhanced green fluorescent protein (EGFP) in infected target cells, thus eliminating the need for antibody staining methods to detect infection efficiency and increased safety due to their replication-deficiency and the apathogenicity of the backbone VSV strain used (Indiana). VSV pseudotypes have been shown to be a safer and faster substitute for authentic PUUV applied in the classical («orthodox») focus reduction neutralization tests (oFRNT), even though the neutralization titers obtained using oFRNT are usually higher than those obtained using the pseudotype Focus Reduction Neutralization Test (pFRNT) [12].

The current therapy for NE is supportive, but there have been reports showing some success with treating hantavirus cardiopulmonary syndrome (HCPS), a related disease caused by orthohantaviruses circulating in the Americas, with immune sera recovered patients [13]. With evolving efforts to clone and harness human neutralizing monoclonal antibodies as a treatment of several emerging infections [14], including hantavirus infections, it is important to ascertain if higher levels of neutralizing antibody responses may help in the effective clearance of viruses or the alleviation of symptoms. To this end, we analyzed the neutralizing antibody levels, PUUV nucleocapsid (N) protein specific antibody levels, and viral RNA loads from the blood samples of a series of patients admitted to hospital with NE and searched for any associations, particularly those between the initial neutralizing antibody titers and the clinical disease severity parameters in NE.

## 2. Materials and Methods

### 2.1. Cells

Vero E6 (African green monkey kidney cells, ATCC CRL-1586) and HEK293T (human embryonic kidney cells, ATCC CRL-11268) were cultivated at 37⁰C, 5% CO_2_, in DMEM supplemented with 5% fetal calf serum (FCS, Gibco, Paisley, UK) and 2 mM L-glutamine. BHK-21 (baby hamster kidney) cell-derived BSRT7/5 cells [15,16], kindly provided by Dr Tero Ahola (University of Helsinki), were grown in similar conditions but supplemented with 1 mg m/L of Geneticin (G418 sulphate, Gibco, Paisley, UK) on every second passage to ensure positive selection of bacteriophage T7 polymerase-expressing cells [16,17]. All cell cultures for rescue, amplification, titration, and pseudotyping process were without antibiotics.

### 2.2. Patient Samples

NE patient samples collected under two different study protocols from September 2000 to March 2009 at the Tampere University Hospital in Finland were used in this study. The study protocols were approved by the Ethical Committee of Tampere University Hospital with permit numbers R04180 and R99256 for 1st (*n* = 55) and 2nd (*n* = 61) set of patient samples, respectively. Written informed consent was received from all patients. For all patients, acute PUUV infection was confirmed serologically [18,19,20]. Routine measurements of plasma creatinine (Cr), blood thrombocyte count (TC), plasma C-reactive protein (CRP), blood leukocyte count (WBC), haematocrit (HCT), and blood pressure were taken daily (excluding weekends) during hospitalization to monitor disease progress. The total duration (Dt) of illness for every patient was calculated as the number of days from the onset of fever to the patients’ discharge. Another variable, fever days before hospitalization (FDBH), was generated as a measure of how long the patients had symptoms before hospitalization. The estimated glomerular filtration rate (eGFR) was calculated using the Chronic Kidney Disease Epidemiology Collaboration (CKD–EPI) equation [21,22,23,24]. Minimum mean arterial pressure (MAPmin) was calculated from each patient’s minimum diastolic and systolic blood pressures during hospitalization, according to an equation by Magder, S.A. (2014) [25]. The overall severity of patients was assessed by an arbitrary severity scale (Sevscale) scoring system adapted from the sequential organ failure assessment system, where the maximum levels of plasma creatinine (4 = > 440, 3 = 300–440, 2 = 171–299, 1 = 110–170, and 0 = < 110 μmol/L), minimum level of thrombocytes (4 = < 20, 3 = 20–49, 2 = 50–99, 1 = 100–150, and 0 = > 150 × 109/L), and a lowest mean arterial blood pressure (MAP) measured during hospitalization (1 = < 70 and 0 = ≥ 70 mmHg) were ranked. The Sevscale was used as an additional measure for disease severity in this study and has not been validated for hantavirus patients. In addition, patient data values for the levels of interleukin 8 (IL-8), myeloperoxidase (MPO), human neutrophil elastase (hNE), histone (H3), citrullinated histone (cH3), and free light chains kappa and lambda (FLCκ and FLCλ), all from the first day of hospitalization, were obtained previously [26,27].

### 2.3. Real-Time RT-PCR

RNA extraction and real-time RT-PCR specific to the PUUV S segment were carried out with plasma obtained at the first day of hospitalization for the 1st sample set (*n* = 55), according to the protocol described by Niskanen et al. [28]. The results were reported as S segment copy numbers using S segment RNA as standard and designated as VL (viral load).

### 2.4. Neutralization Assay (pFRNT)

Replication-deficient recombinant VSV-PUUV pseudotypes were produced, and the pFRNT assay performed from the patients’ serum samples on the first day of hospitalization using previously described protocols [12]. The strain of PUUV (Sotkamo, Finland), which the pseudotyping assay is based on, is one of the strains originating in Finland and is very closely related (serologically) to other domestic strains [12]. Approximately 100 infectious units of PUUV-VSV pseudotypes were mixed with an equal volume of serially diluted serum samples. Serial dilution was made in 2-fold steps starting from the seropositive threshold titer 1/40 to give a more precise estimation of the end point titer. PUUV neutralizing monoclonal antibody 4G2 [29] or serum-free media were used as a positive control or negative control, respectively. After a 2 h incubation at 37 °C, 50 µL was used to inoculate 50,000 Vero E6 cells plated and grown overnight in a 96-well culture plate (Nunc, Roskilde, Denmark). After 45 min, the inoculum was replaced with 100 µL of DMEM supplemented with 5% FCS and the plate incubated at 37 °C for 16 h. EGFP expressing cells were enumerated under a blue 475 filter on the PerkinElmer Opera Phenix high content imager (PerkinElmer, Waltham, MA, USA), and the neutralizing (end-point) titers were computed as the reciprocal of the serum dilution that resulted in an 80% reduction in fluorescent foci as compared to the mean of the virus controls (without serum or neutralizing MAbs). The result of this assay was designated NAb.

### 2.5. PUUV Nucleocapsid Protein (PUUV-N) Antibody ELISA

ELISA (enzyme linked immunosorbent assay) was done using a modified protocol published by Vapalahti et al. [20]. ELISA plates (Nunc, Roskilde, Denmark) were coated with baculovirus-expressed, guanidine-HCl-purified, PUUV-N at a concentration of 5 µg/mL in 0.05M carbonate-bicarbonate buffer pH 9.6 (Medicago, Uppsala, Sweden) 100 µL/well of overnight at +4 °C. The next day, the wells were blocked for 30 min at 37 °C with 150 µL of blocking buffer (1% bovine serum albumin in PBS) and washed once with PBS-T (PBS and 0.05%Tween 20), and 100µL of PBS-T diluted patient sample was added, incubated at 37 °C for 45 min, and washed five times with PBS-T. After washes, the secondary antibody, Polyclonal Rabbit Anti-Human IgG, IgM, or IgA (all conjugated with horseradish peroxidase, HRP, Dako, Glostrup, Denmark), diluted by manufacturer’s specifications, was added (100 µL/well); the plate was incubated for 30 min at 37 °C and washed 5 times with PBS-T, followed by the addition of the substrate 3,3′,5,5′-Tetramethylbenzidine (TMB; Sigma, Rockford, IL, USA) 100 µL/well and 20 min incubation at room temperature. The reaction was terminated by the addition of 0.5 M sulphuric acid solution (Honeywell, Germany) 50 µL/well, and the absorbance was read on the HIDEX sense microplate reader with the software version 0.5 at a wavelength of 450 nm. The optical density (OD) in each well was reported for patient samples after the subtraction of a signal obtained from wells without a patient sample and designated as PUUV–N–IgG.

### 2.6. Statistical Analysis

#### 2.6.1. Preliminary Data Processing

We chose 24 variables to be included in our statistical analyses. The data were checked for outliers using the interquartile range rule and a multiplier of 2.2 [30], and the normality check for each variable was done with Shapiro–Wilk test. Our data were mostly categorical or non-normal, and we preferred non-parametric and non-normal methods in our analyses. We named the entity of 24 variables as the “basic dataset” (Table S1b), and it had observations from 116 patients. We further removed the observations that contained missing values from the basic dataset and scaled the variables between zero and one. (Table S1a). This entity was called the “scaled dataset”, and it had observations from 49 patients. Wilcoxon rank sum tests were used to test the similarities of distributions between the basic and scaled datasets. We counted the descriptive statistics—distributions, frequencies, means, and ranges—of the variables and used Spearman correlations to determine the associations between the variables. *p*-values of less than 0.05 of ρ (correlations) defined statistically significant correlation.

#### 2.6.2. Associative Analyses Using Kruskal–Wallis and General Linear Models

The association between the PUUV–NAb and NE disease severity markers was examined in the Kruskal–Wallis tests. Patients were stratified, based on their PUUV–NAb titers, into four categories: NAb ≤ 320, NAb = 640, NAb = 1280, and NAb ≥ 2560. In the tests, categorical NAb was the dependent variable, and all other variables individually served as the independent variable. The test yielded the mean squares of the independent variable in the categories of NAb. A *p*-value of less than 0.05 of the chi-square test statistic indicated a significant difference between mean ranks in the NAb1 categories.

We utilized the general linear model (GLM) to examine the associations between VL, eGFRmin, eGFR, PUUV–N–IgG, and all other variables. GLM models were generated using either VL, eGFRmin, eGFR, or PUUV–N–IgG as a dependent variable, and all other variables individually as an independent variable. FDBH was included in all these models as a confounding factor to control its possible effect on the independent variable. The model estimate indicated the dependent variable’s change with respect to the unit increase in the independent variable. *p*-values less than the 0.05 *F*-value defined a statistically significant association.

#### 2.6.3. Time Series Analysis and Hierarchical Cluster Analysis

We had a dataset of the patients’ eGFR, haematocrit (HCT), leukocyte (WBC), platelet (TC), and CRP values for the first four days of hospitalization. A time series of eGFR, HCT, WBC, TC, or CRP were examined in different categories of NAb, VL, and PUUV–N–IgG. The NAb titers were divided into 4 categories: Nab < 320, Nab = 640, Nab = 1280, and NAb1 ≥ 1280. VL had 3 categories: VL < 10, 10 ≤ VL < 80, and VL ≥ 80. The 3 categories of PUUV–N–IgG were < 0.15, 0.15 ≤ PUUV–N–IgG < 0.9, and PUUV–N–IgG ≥ 0.9. In the analyses, we utilized mixed models with either eGFR, HCT, WBC, TC, or CRP as the dependent variable, and time, one of the categorized variables, and their interaction serving, as the independent variables. The independent variable’s p value of less than 0.05 denoted statistical significance, which means that the independent variable explains a significant proportion of the variance of the dependent variable. The estimate sign showed the direction of the association. There were 54 patients in the time series.

We performed the hierarchical cluster analysis in the scaled dataset using HCTmin, MAPmin, TCmin, VL, PUUV–N–IgG, Nab1, IL-8, FLCκ, WBCmax, MPO, FDBH, hNE, Dt, and eGFRmin variables. We used the Euclidean distance as the distance metrics, and Ward’s linkage method in clustering. The silhouette plot was used in the evaluation of the clustering. The heatmap figure was created based on the best clustering, as defined by the highest average silhouette width.

#### 2.6.4. Statistical Software

Descriptive statistics were tabulated using SPSS 24.0 [31]. SAS© 9.4 proc glm and proc mixed procedures were used in general linear and mixed modelling, respectively, whereas the normality test and distribution analyses were done using the SAS© 9.4 proc univariate procedure with the normal option and Wilcoxon option, respectively. The SAS© 9.4 proc npar1way procedure with the Wilcoxon option was used in the Kruskal–Wallis analysis [32].

The R studio was used for the calculation and plotting of the correlations (Hmisc and corrplot packages), while the hierarchical cluster analysis, heatmaps, and silhouette plot were done by R cluster and pheatmap packages, respectively [33].

## 3. Results

### 3.1. Patient Characteristics

There are 116 (66% male and 34% female) hospitalized patients with acute NE in this study. The descriptive clinical and laboratory parameters are listed in Table S1. There were no fatal cases, although five patients received dialysis treatment due to acute kidney injury (AKI), the main manifestation of severe NE. Patients were admitted to hospital on average 5 days after onset of fever ranging from 1–15 days. The mean duration of illness, measured from time of onset of fever to discharge from hospital (Dt), was 11 days, ranging between 4 and 21 days. We quantified the extent of AKI by assessing estimated glomerular filtration rate (eGFR) during hospitalization. The average minimum eGFR for the hospital stay was 65.23 mL/min/1.73 m^2^, ranging from 4 to 128 mL/min/1.73 m^2^. As a marker of overall disease severity, we calculated a severity value (Sevscale) for each patient (see materials and methods for details). The average Sevscale value for the patients was 3, ranging between 0 and 8. The patients were classified into severe (Sevscale > 3) and mild (Sevscale ≤ 3) with 40 and 76 patients, respectively. Since the patient data (data with all patients was named basic dataset) included several missing values, we performed parallel statistical analyses excluding patients with missing values (scaled dataset with 49 patients, 59% male, and 41% female). In the hierarchical cluster analysis, we were exceptionally able to utilize the scaled data of 51 patients instead of 49 (60% male and 40% female) since variables in the final heatmap included fewer missing values than the variables of the whole dataset. The two added patients were males with mild disease. The results presented below are based on basic data unless stated otherwise.

**Figure 1:**
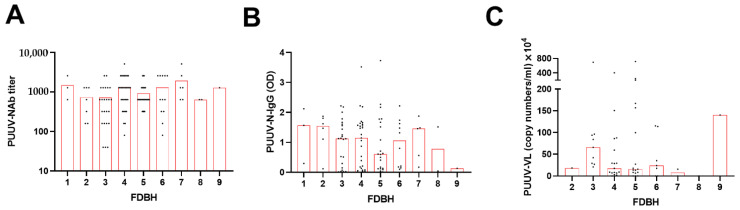
Graphs showing (**A**) PUUV neutralizing antibody titer, (**B**) PUUV nucleocapsid (N) protein-specific IgG, and (**C**) PUUV viral load from serum at first day of hospitalization as assessed by duration of fever days before hospitalization (FDBH). OD = optical density. The bar indicates median values.

All patients displayed detectable circulating neutralizing antibodies (PUUV–NAb) and N protein-specific IgG (PUUV–N–IgG) in serum samples taken on the first day of hospitalization (Figure 1A,B). There was no evident trend between PUUV–NAb titers and fever days before hospitalization (FDBH), whereas higher N-specific IgG levels seemed to be associated with shorter FDBH. In terms of circulating viral RNA (VL), 45 out of 53 patients had detectable viral RNA in serum but without showing an obvious trend in comparison to FDBH (Figure 1C).

### 3.2. Pairwise Correlations between PUUV Neutralizing and Nucleocapsid Protein-Specific Antibody Levels, Viral Load, and Disease Severity Parameters

**Figure 2:**
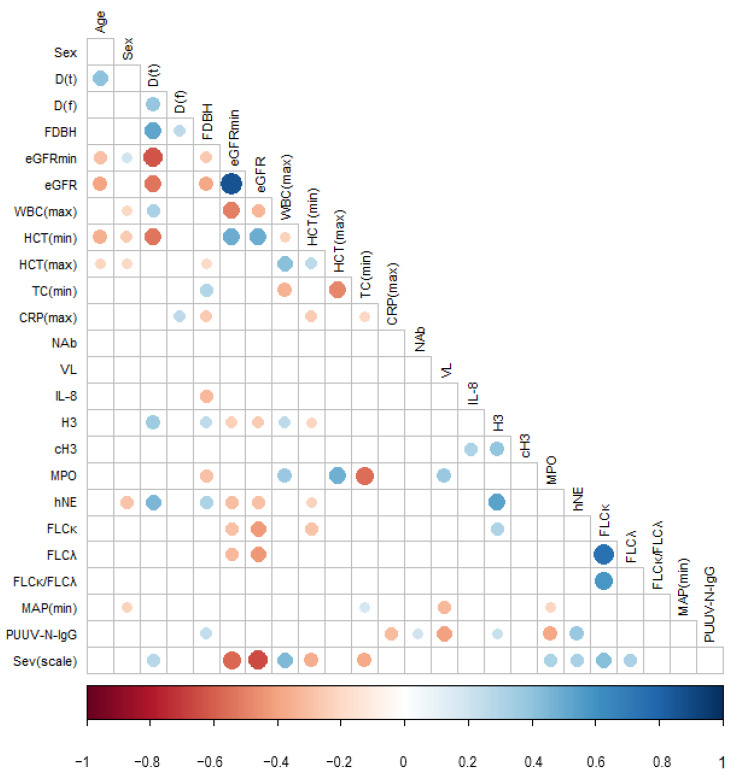
The Spearman correlation plot of the basic dataset where missing data points in variables were excluded pairwise. Variable abbreviations are D(t) = overall disease duration, D(f) = overall duration of fever, FDBH = fever days before hospitalization, eGFR = estimated glomerular filtration rate, WBC = white blood cell count, HCT = hematocrit, TC = thrombocyte count, CRP = C-reactive protein, NAb = PUUV neutralizing antibodies, VL = PUUV viral load, IL-8 = interleukin-8, H3 = histone H3, cH3 = citrullinated histone H3, MPO = myeloperoxidase, hNE = neutrophil elastase, FLCκ = free light chain κ, FLCλ = free light chain λ, MAP = mean arterial pressure, and Sev(scale) = severity scale. The maximum or minimum values during the hospitalization are indicated as min or max, respectively, and all other parameters are measured from the first day of hospitalization. Only significant correlations at *p* ≤ 0.05 are shown, and the size of the dots is equivalent to the significance. Note that the significance of Sevscale for eGFR and eGFRmin is redundant due to the shared parameters defining them.

We performed pairwise correlation of the currently measured parameters PUUV–NAb, PUUV–N–IgG, and PUUV–VL, all from the first day of hospitalization, with a set of clinical and laboratory parameters analyzed from the same patients. The parameters were measured at the first day of hospitalization if they were not the minimum (min) or maximum (max) value recorded during the hospital stay (marked as e.g., eGFRmin). Perhaps expectedly, neutralizing antibody titers upon hospitalization correlated with PUUV N protein IgG antibodies from the same day (ρ (99) = 0.208, *p* = 0.039). However, no significant correlations between PUUV NAbs and disease severity markers were observed (Figure 2 and Table S3). Patients with higher PUUV–N–IgG antibody levels had significantly lower maximum C-reactive protein CRPmax (ρ (98) = −0.317, *p* = 0.001) and myeloperoxidase MPO (ρ (79) = −0.386, *p* < 0.001) but higher circulating neutrophil elastase hNE (ρ (76) = 0.372, *p* = 0.001) and histone H3 (ρ (85) = 0.224, *p* = 0.039). As already suggested by Figure 1B, PUUV–N–IgG also correlated positively with FDBH (ρ (99) = 0.241, *p* = 0.017).

Importantly, viral load (VL) correlated negatively with PUUV–N–IgG (ρ (55) = −0.410, *p* = 0.002). It also correlated positively with MPO (ρ (53) = 0.373, *p* = 0.006) and negatively with minimum mean arterial pressure MAPmin (ρ (55) = −0.326 *p* = 0.015). There were no other significant associations between the viral load and other measured clinical markers (Figure 2 and Table S3). The basic and scaled datasets provided essentially comparable results in pairwise correlations (Figure S1 and Tables S2 and S3).

### 3.3. Extended Statistical Analysis of the Association between PUUV–NAb1, PUUV–N–IgG1 and PUUV–VL1, and Disease Severity

In order to further understand the association between the PUUV–NAb and NE disease severity markers, patients were stratified based on their PUUV–NAb titers into four categories: NAb ≤ 320, NAb = 640, NAb = 1280, and NAb ≥ 2560, and the distribution of different severity parameters between these categories was assessed using Kruskal–Wallis tests. The analysis showed a significant association between PUUV–NAb and VL (Figure S2). However, except for citrullinated histone H3 (cH3) and myeloperoxidase (MPO), none of the variables of interest were associated with PUUV–NAb (Table S4).

The association of the continuous variables PUUV–IgG and PUUV–VL with disease severity parameters were addressed through general linear models (GLMs). We also included eGFR from the first day of hospitalization (eGFR) and the minimum value observed through hospitalization (eGFRmin) as individual dependent variables in the GLM analysis. PUUV–N–IgG was significantly associated with neutrophil markers MPO, as well as with CRPmax, whereas PUUV–VL had a significant positive association only with the FLCκ/FLCλ ratio. Surprisingly, eGFR had a significant negative association with PUUV–NAb, which suggested that higher NAb levels are associated with more severe kidney injury upon admission. As expected, eGFR and eGFRmin were associated with several parameters reflecting disease severity, including Dt, WBCmax, hNE, FLCκ, FLCλ, and HCTmin, as well as age and FDBH (Table S4). It is worth noting that the significance of Sevscale for eGFR and eGFRmin is redundant due to the shared parameters defining them. When comparing the results in the basic and scaled datasets, the GLM and Kruskal–Wallis analyses provided essentially comparable results (Table S4).

### 3.4. Time Series Analysis

**Figure 3:**
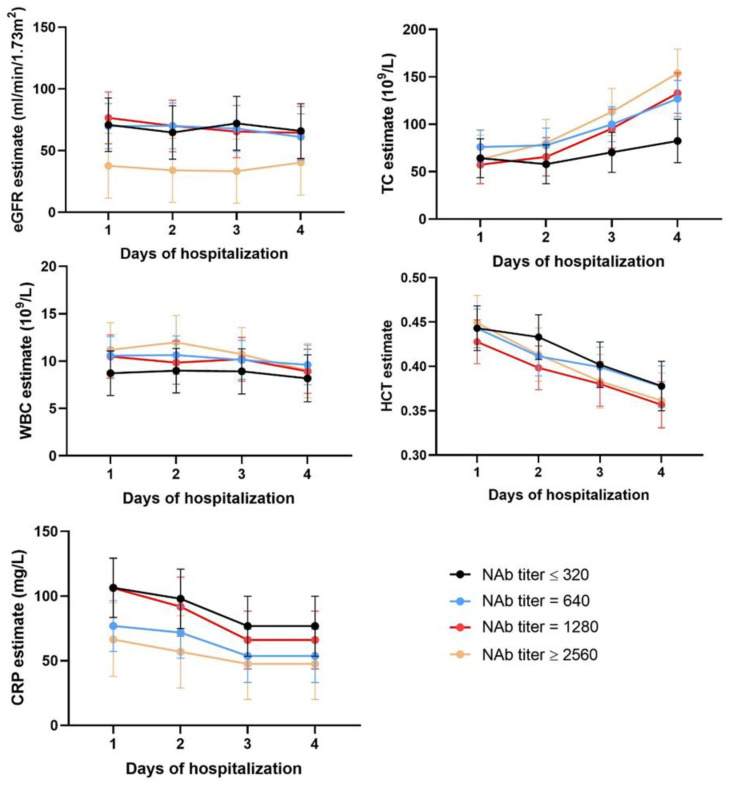
Time-series graphs showing the impact of different categories of NAb on the first day of hospitalization on the estimated glomerular filtration rate (eGFR), blood thrombocyte count (TC), white blood cell count (WBC), haematocrit (HCT), and C-reactive protein (CRP). The variables were followed over time (the first 4 days of hospitalization) and reported as mean estimates obtained from the mixed model analysis. The error bars indicate upper and lower limits of the estimates.

To further evaluate the predictive value of PUUV–NAb, PUUV–N–IgG, and PUUV–VL in NE disease severity, eGFR, haematocrit (HCT), the white blood cell count (WBC), thrombocytes (TC), and the C-reactive protein (CRP) were followed over time during the initial four days of hospitalization. Patients were stratified into 3 or 4 categories based on their level of PUUV–NAb, PUUV–N–IgG, or PUUV–VL upon the first day of hospitalization. Interestingly, patients with the highest PUUV–NAb levels seemed to have lower eGFR levels (Figure 3), although this was not statistically significant in a mixed model analysis (Table S4). However, PUUV–NAb in interaction with time had a significant effect on the variation of thrombocyte counts (TC) (*p* = 0.003), whereas it had no effect in the variation of other dependent variables (Table S5). Based on Figure 3, it was apparent that, in patients with higher PUUV–NAbs, thrombcyte counts normalized at a faster rate during hospitalization. Along the same lines, the interaction of PUUV–N–IgG with time had a significant effect on the variation of TC (Table S5 and Figure S3), whereas PUUV–VL did not interact with any of the independent variables (Table S5 and Figure S4).

### 3.5. Hierarchical Cluster Analysis

**Figure 4:**
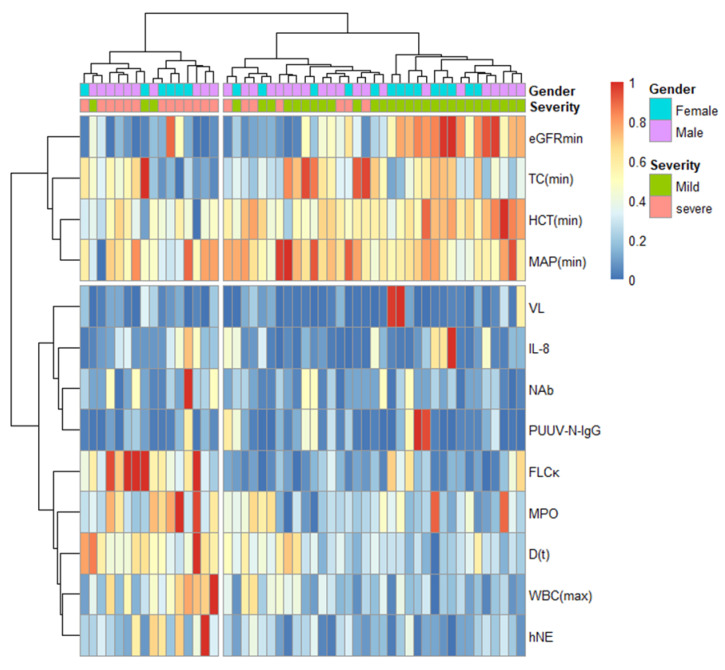
A heatmap of hierarchical clustering analysis using scaled data (*n* = 51). Each column represents a patient. Severity was assessed based on Sevscale (mild ≤ 3 and severe > 3). The unsupervised hierarchical clustering divided the variables in two main groups, with the patients grouped to the left being seemingly more severe (based on Sevscale) and patients to the right seemingly milder. The average silhouette distance of the hierarchical clustering was 0.25. Note that eGFR and severity are closely related due to shared parameters in their calculation.

To visualize the spread in the level of PUUV–NAb, PUUV–N–IgG, and PUUV–VL in patients with NE of varying severity, 13 variables were included in an unsupervised cluster analysis. The best clustering according to silhouette plot was gained by dividing the variables into two main groups in the analysis (Figure 4, silhouette plot shown in Figure S5). As expected, the patients within the group with apparently more severe NE (patients clustered to the left in Figure 4) seemed to have higher levels of WBCmax and neutrophil activity (as seen from elevated MPO, hNE and IL-8) and lower eGFRmin and MAPmin. On the other hand, the patients within the mild group seemed to have higher eGFRmin and HCTmin. Importantly, of the parameters measured in this study, PUUV–NAb, PUUV–N–IgG, and PUUV–VL, none were seemingly associated with either mild or severe NE clusters.

## 4. Discussion

The treatment of nephropathia epidemica (NE), as well as other hantavirus infections, has been mainly supportive, although in some cases of severe HCPS, immune sera has been used to treat patients [13]. In addition, immune sera and neutralizing monoclonal antibody (nMAb) treatments have been shown to be effective post-exposure prophylactics for the prevention of severe outcomes in experimental PUUV infections in cynomolgus macaques [34,35]. However, results from studies on the use of the antiviral drug ribavirin as a therapeutic agent for HFRS and NE have been discrepant [36]. Good outcomes with the post-exposure administration of ribavirin have been seen for HFRS in China [37], while studies for NE showed that ribavirin administered within 4 days of the onset of symptoms was ineffective in reducing symptoms and altering viral infection kinetics [38]. However, the current consensus is that ribavirin use is ineffective after the onset of symptoms [36]. In light of this, we sought to inform approaches based on using neutralizing antibodies in the treatment of NE patients first by ascertaining if a high initial neutralizing antibody titer is a prognostic factor in NE. Contrary to the assumption that higher initial neutralizing antibody titers should lead to better prognosis, we did not observe any association between PUUV NAbs and disease course. Although we need to keep in mind that therapeutically given “external” antibodies may play a different role than those developing in the diseased patient [39], these results are not directly supportive of the therapeutic potential of immune sera or nMAbs in hospitalized NE patients. Our results suggest that for the PUUV neutralizing antibody therapy to potentially benefit NE patients, it likely should be administered as early as possible (if not as post-exposure prophylaxis) and because, typically, severe patients had longer FDBH to the point that viremia was decreased or gone; therefore, the disease process is probably being fanned by a hyperstimulated immune response. This is also in line with the consensus idea of immune-mediated pathogenesis in HFRS. However, our data were limited to hospitalized patients (no mild outpatient or subclinical cases) and did not start from the first days of illness, so the potential of the antibody therapy in NE remains to be elucidated in further studies. These will also be needed to understand whether the success of immune serum treatment is unique to HCPS and not applicable to HFRS of different severities. Different pathological mechanisms and variable infection kinetics between HCPS and HFRS should be considered when assessing the outcome of therapeutic trials aimed at dampening viral loads in patients with orthohantavirus infections.

Despite not directly correlating with any other measured variable with the exception of PUUV-N IgG antibodies, PUUV neutralizing antibody titers were associated with severe AKI upon hospitalization by GLM analysis and showed a trend of higher levels in patients with more significantly impaired renal function (lower eGFR) in the time series analysis. This is contrary to what has been reported for infections with Sin Nombre orthohantavirus [40], which causes a different disease phenotype (HCPS) with a different organ targeting (lungs instead of kidneys). Interestingly, our previous results have shown that severe NE patients have higher circulating free light chain levels [41], which are considered as markers of polyclonal B cell activation and increased total humoral responses. This serves as a speculative support for the observed higher neutralizing antibody levels, being part of a general increase in antibody production in severe NE cases. Importantly, while similarly upregulated, free light chains do not correlate with disease severity in acute HCPS [41], further suggesting variable pathogenic mechanisms between the two orthohantaviral diseases in which increased humoral response might be contributing to NE (and HFRS) pathogenesis, whereas HCPS would be dominated by direct virus infection-related mechanisms. It should be noted that this lower level of eGFR in patients with higher NAb is likely not due to immunocomplex deposition in kidneys since previous works on the matter had shown no evidence of such complexes by immunofluorescence methods in kidney biopsies [42].

As with PUUV–NAbs, we did not detect a strong correlation between PUUV N-specific IgG levels and disease severity, although N-specific IgG was associated with neutrophil activity markers in GLM analysis. Contrary to our findings, an association between low PUUV-specific IgG titers and disease severity has been reported previously by a group of Swedish NE patients [43], in which they measured IgG antibody responses against all virus proteins using PUUV-infected cells as the target. The PUUV NAbs represent the Ab response to viral surface glycoproteins, the kinetics of which can differ from those targeting the N protein and explain at least some of the discrepant results between our different analysis methods. The other possibility is variation in the severity classification between our current study and the abovementioned study from Sweden. We emphasize that AKI is the most important manifestation of PUUV severity, whereas in the previous study, kidney dysfunction was not considered as a defining parameter of disease severity [43].

It is of interest to note that both PUUV–NAb and PUUV–N–IgG levels were positively associated with the faster recovery of blood thrombocyte counts in a time series analysis. This suggests that virus-specific humoral responses would clear the infection more rapidly from circulation and infected endothelial cells, thus facilitating the faster recovery from vasculopathological mechanisms leading to thrombocytopenia. In this scenario, the pathological mechanisms leading to AKI would be detached from direct virus-infection-related pathology in the vasculature. This is in line with our previous reports of a lack of association between AKI and thrombocytopenia [44]. This is highly hypothetical, however, and one can appreciate the potential of virus-infected glomerular endothelial cells as drivers of diminished eGFR and worsened AKI.

As expected, lower viral load (as indicated by viral RNA in circulation) was associated with higher NAbs and PUUV–N–IgG levels. The lack of association between viral load and pathophysiology of severe NE supports the idea of virus-initiated exacerbation of immune activation, but not direct virus infection levels, as the driving force of NE pathology. However, it needs to be kept in mind that serum viral load decreases rapidly in NE patients [45] and is a relatively poor estimate of viral replication at the initial phase of NE. In fact, the steep decline of viral RNA upon hospitalization implies significantly higher viral loads in the early phases of the disease, and the real extent of the virus replication prior to and directly after the fever debut are still unclear.

In the absence of a consensus agreement on the criteria for the severity of NE, we used eGFR as the main marker of AKI since AKI is traditionally diagnosed using change in serum creatinine and or urinary output [46]. Although eGFR is accurate to assess kidney function during chronic kidney injury, its use during AKI is controversial because of its fast onset and resolution. However, we decided to use eGFR instead of serum creatinine (sCr) as an indication of AKI in this study, since eGFR takes the gender and age of the patients into account and also adjusts for patients’ body surface area, all of which affect sCr readings. Although albuminuria is a useful measure of kidney function, it is not required for the traditional definition of AKI [46] and was not available to us. In addition, we also calculated a severity scale (Sevscale) for the patients based on blood creatinine, minimum mean arterial pressure, and thrombocyte count in order to group the patients into severe and mild cases and noted that this correlated positively with duration of illness, which can be considered as another marker of disease severity. While we included mean arterial pressure (MAP) as a factor in Sevscale calculations, we are aware that hemodynamic failure and significant hypotension are not major manifestations in the pathogenesis of NE. In fact, the contribution of MAP to the Sevscale calculation is negligible.

The samples used in this study were archival samples collected under two separate study protocols. Differences in the parameters measured from the two datasets posed limitations for the current statistical analysis by introducing missing values. This was partially overcome by using a scaled dataset, the results of which suggested that the exclusion of missing values did not alter distributions critically. In addition, our assessment of virus-specific Ab responses was based solely on N-specific IgG and neutralizing antibodies but did not include non-neutralizing Abs against viral glycoproteins. In order to fully understand the total humoral responses that contribute to PUUV neutralization, glycoprotein-specific Abs of different isotypes also need to be assessed. In addition, this study involved only hospitalized patients, and future studies would benefit from including outpatients, who consist 50% of all diagnosed cases [47].

## 5. Conclusions

Altogether, our results show that the level of NE patient early (from the first day hospitalization day) neutralizing antibody response as measured from the first sample in hospital cannot be correlated with the clinical outcome or disease severity. While this may be affected by, e.g., the avidity or antibody class, and the HFRS and HCPS type hantavirus illnesses may have different kinetics and play different roles in neutralizing antibodies, further studies are needed to ascertain the therapeutic value of possible neutralizing monoclonal antibody therapies in the development of the treatment of NE and other hantaviral diseases.

## Data Availability

The data presented in this study are available on request from the corresponding author. The data are not publicly available due to other ongoing and related works involving the data.

## Supplementary Materials

The following supporting information can be downloaded at: https://www.mdpi.com/article/10.3390/v14050901/s1, Figure S1: a Spearman correlation plot of scaled data; Figure S2: a time series-graph showing the impact of different levels of PUUV–N–IgG on the first day of hospitalization on eGFR, TC, WBC, HKR, and CRP; Figure S3: a time-series graph showing the impact of different levels of PUUV VL on the first day of hospitalization on eGFR, TC, WBC, HKR, and CRP; Figure S4: a silhouette plot; Figure S5: Silhouette plot of hierarchical cluster analysis. Average silhouette width 0.25. Cluster 1: 9 clusters, average silhouette width 0.27. Cluster 2: 4 clusters, average silhouette width 0.20. The clustering with the highest average silhouette width is considered to present the most appropriate clustering of the data points. Table S1: descriptive statistics; Table S2: a pairwise Spearman correlation table; Table S3: *p*-values for a pairwise Spearman correlation; Table S4: a Kruskal–Wallace and general linear model analysis; Table S5: a mixed model analysis.
