# Supplementary material for: Neutralizing Antibody Titers in Hospitalized Patients with Acute Puumala Orthohantavirus Infection Do Not Associate with Disease Severity
Source: Viruses. 2022 Apr 26;14(5):901. doi: 10.3390/v14050901 (PMC9143849; doi:10.3390/v14050901)
Supplement: Supplementary file 1 [file viruses-14-00901-s001.zip › Supplementary materials (004)_KV19042022.pdf]

viruses

MDPI

High early neutralizing antibody titres in patients with acute Puumala orthohantavirus infection do not associate with reduced disease severity

Rommel Iheozor-Ejiofor[1]* #, Katariina Vapalahti[4], Tarja Sironen[1,2], Lev Levanov[1], Jussi Hepojoki[1,3], Åke Lundkvist[3], Satu Mäkelä[7,8], Antti Vaheri[1], Jukka Mustonen[7,8], Alexander Plyusnin[1,3], Tomas M. Strandin[1]*and Olli Vapalahti[1,2,4]

Supplementary materials

Table S1. Descriptive statistics table showing clinical and laboratory parameters of NE patients included in the study. Variable abbreviations are Dt = Overall disease duration, Df = Fever duration, FDBH = Fever days before hopsitalization, eGFR = estimated glomerular filtration rate, WBC = white blood cell count, HCT = Hematocrit, TC = thrombocyte count, CRP = C-reactive protein, NAb = PUUV neutrlalizing antibodies, VL = PUUV viral load, IL-8 = Interleukin-8, H3 = Histone H3, cH3 = Citrullinated histone H3, MPO = Myeloperoxidase, hNE = Neutrophil elastase, FLCκ = Free light chain κ, FLCλ = Free light chain λ, MAP =mean arterial pressure, Sev(scale) = Severity scale. A) Scaled data with all missing data excluded B) the basic data.

(A) Scaled Data Descriptive Statistics

	Age	sex	FDBH	Df	Dt	WBCmax	HCTmin	TCmin	CRPmax	Nab	VL
N Valid	49	49	49	49	49	49	49	49	49	49	49
N Missing	0	0	0	0	0	0	0	0	0	0	0
Normal range	NA	NA	NA	NA	NA	11 x 10^9/L	0.36-0.41	150x10^3/µL	95.2nmol/L	NA	NA
Mean	42,74		4,57	8,12	9,63	11,39	0,35	59,71	97,58	1020	84,95
Median	40,80	0	4,00	8,00	9,00	10,30	0,36	53,00	91,60	640	26,81
Mode	27.50^a	0	4,00	8,00	8,00	8,80	0,36	35^a	91,60	640	0,00
Range	51,10		7,00	11,00	14,00	18,40	0,19	103,00	250,90	5040	717,50
Minimum	22,40	Male = 0	2,00	4,00	4,00	5,60	0,25	15,00	15,90	80	0,00
Maximum	73,50	Female = 1	9,00	15,00	18,00	24,00	0,44	118,00	266,80	5120	717,50

	IL8	H3	cH3	MPO	FLCk/FLCλ	MAPmin	SEVscore	PUUV-N-IgG	hNE	FLCk	FLCλ	Htmax	eGFRmin	eGFR
N Valid	49	49	49	49	49	49	49	49	49	49	49	49	49	49
N Missing	0	0	0	0	0	0	0	0	0	0	0	0	0	0
Normal range	≤0.062ng/mL	0.2-2.7ng/ml	866-1887ng/dL	≤285ng/mL	0.26-1.65	≥60mmHg	NA	NA	74-413ng/ml	3.3-19.4mg/L	5.71-26.3mg/L	0.45-0.49	≥60ml/min/1.73m	≥60ml/min/1.73m^2
Mean	0,28	5,70	336,06	157,57	1,16	83,74	3	0,57	115,36	34,19	29,56	0,44	49,88	67,53
Median	0,13	4,79	325,73	131,59	1,04	83,33	3	0,17	98,71	24,44	24,26	0,44	38,00	73,00
Mode	.020^a	4,72	214.690^a	126.490^a	.35^a	92,00	2	.010^a	57.280^a	87,02	24,04	0,44	5.00^a	7.00^a
Range	1,45	20,07	379,89	286,13	2,19	52,00	7	3,73	419,27	77,72	62,54	0,26	120,00	120,00
Minimum	0,00	1,16	214,69	46,04	0,35	51,33	0	0,00	11,84	9,30	8,80	0,33	3,00	7,00
Maximum	1,45	21,23	594,58	332,17	2,55	103,33	7	3,73	431,11	87,02	71,34	0,59	123,00	127,00

a. Multiple modes exist. The smallest value is shown

(B) Basic Data Descriptive Statistics

	Age	sex	FDBH	Df	Dt	WBCmax	HCTmin	TCmin	CRPmax	Nab	VL
N Valid	116	116	116	114	114	115	115	114	115	116	55
N Missing	0	0	0	2	2	1	1	2	1	0	61
Mean	44,21		4,41	8,17	10,68	11,48	0,36	68,80	92,43	1082	77,62
Median	41,10	0	4,00	8,00	10,00	10,00	0,36	61,00	80,70	640	23,88
Mode	27.50^a	0	4,00	8,00	8,00	8.800^a	0,36	50,00	91,60	640	0,00
Range	55,30		14,00	15,00	17,00	27,30	0,19	229,00	253,30	5080	717,50
Minimum	21,50	Male = 0	1,00	3,00	4,00	3,90	0,25	9,00	15,90	40	0,00
Maximum	76,80	Female = 1	15,00	18,00	21,00	31,20	0,44	238,00	269,20	5120	717,50

	IL8	H3	cH3	MPO	FLCk/FLCλ	MAPmin	SEVscore	PUUV-N-IgG	hNE	FLCk	FLCλ	Htmax	eGFRmin	eGFR
N Valid	52	93	52	85	55	115	116	99	83	55	55	114	116	110
N Missing	64	23	64	31	61	1	0	17	33	61	61	2	0	6
Mean	0,27	8,32	335,83	135,51	1,16	84,66	3	1,01	148,71	34,04	29,31	0,44	49,31	65,23
Median	0,13	6,75	322,81	117,39	1,06	84,67	3	1,11	131,80	24,44	24,26	0,44	41,00	72,00
Mode	.020^a	4,72	214.690^a	126.490^a	.35^a	92.00^a	2	.010^a	57.280^a	87,02	24,04	0,44	7,00	7,00
Range	1,45	28,59	379,89	286,13	2,19	71,34	8	3,73	517,53	77,72	62,54	0,26	124,00	124,00
Minimum	0,00	1,16	214,69	46,04	0,35	49,33	0	0,00	11,84	9,30	8,80	0,33	3,00	4,00
Maximum	1,45	29,75	594,58	332,17	2,55	120,67	8	3,73	529,37	87,02	71,34	0,59	127,00	128,00

a. Multiple modes exist. The smallest value is shown

Table S2. Spearman correlation table for all the variables for both basic and scaled data. For abbreviations, see Sup-plementary Table S1.

	Variable	Age	sex	D(t)	D(f)	FDBH	eGFR(min)	eGFR	WBC(max)	HCT(min)	HCT(max)	TC(min)	CRP(max)	NAb	VL	IL-8	H3	cH3	MPO	hNE	FLCκ	FLCλ	FLCκ/FLCλ	MAP(min)	PUUV-N-IgG	Sev(scale)
**Basic dataset**	Age		0.019	0.402	0.096	0.165	-0.297	-0.395	-0.020	-0.357	-0.213	0.161	0.176	-0.073	0.084	-0.115	0.097	0.151	-0.018	0.050	0.207	0.198	0.093	-0.106	-0.085	0.106
	sex	0.019		-0.082	0.015	-0.080	0.197	0.115	-0.205	-0.257	-0.209	-0.134	0.025	0.157	0.057	0.123	-0.142	0.184	0.065	-0.290	-0.058	-0.089	0.036	-0.229	-0.060	-0.076
	D(t)	0.402	-0.082		0.388	0.523	-0.624	-0.537	0.324	-0.533	-0.025	-0.047	0.064	0.045	0.024	0.026	0.351	-0.039	-0.021	0.453	0.232	0.252	0.045	-0.047	0.175	0.283
	D(f)	0.096	0.015	0.388		0.265	-0.026	0.054	0.018	-0.133	0.007	0.013	0.269	-0.041	0.153	-0.177	0.047	-0.023	0.039	-0.002	-0.039	-0.056	-0.045	-0.102	-0.030	-0.106
	FDBH	0.165	-0.080	0.523	0.265		-0.265	-0.389	-0.019	-0.111	-0.193	0.293	-0.261	0.117	-0.207	-0.324	0.260	-0.017	-0.290	0.318	0.174	0.185	0.001	0.095	0.241	0.062
	eGFR(min)	-0.297	0.197	-0.624	-0.026	-0.265		0.870	-0.504	0.495	-0.026	0.028	0.031	-0.027	0.093	0.049	-0.239	0.057	-0.145	-0.307	-0.296	-0.322	-0.119	-0.081	-0.036	-0.577
	eGFR	-0.395	0.115	-0.537	0.054	-0.389	0.870		-0.336	0.500	0.094	-0.025	0.107	-0.119	0.080	0.101	-0.260	0.000	-0.039	-0.298	-0.427	-0.430	-0.191	-0.021	-0.033	-0.652
	WBC(max)	-0.020	-0.205	0.324	0.018	-0.019	-0.504	-0.336		-0.225	0.410	-0.342	0.032	-0.040	0.032	0.085	0.262	-0.064	0.374	0.211	0.239	0.107	0.186	-0.081	0.097	0.447
	HCT(min)	-0.357	-0.257	-0.533	-0.133	-0.111	0.495	0.500	-0.225		0.264	0.178	-0.251	-0.043	0.066	0.125	-0.219	0.071	-0.017	-0.226	-0.284	-0.256	-0.114	0.138	0.025	-0.362
	HCT(max)	-0.213	-0.209	-0.025	0.007	-0.193	-0.026	0.094	0.410	0.264		-0.481	-0.082	-0.015	0.121	0.132	0.083	0.176	0.481	0.015	0.092	0.011	0.068	-0.051	0.073	0.127
	TC(min)	0.161	-0.134	-0.047	0.013	0.293	0.028	-0.025	-0.342	0.178	-0.481		-0.202	0.002	-0.259	-0.111	-0.003	-0.205	-0.551	-0.068	-0.196	-0.110	-0.098	0.186	0.116	-0.375
	CRP(max)	0.176	0.025	0.064	0.269	-0.261	0.031	0.107	0.032	-0.251	-0.082	-0.202		-0.082	0.184	-0.033	-0.010	-0.164	0.090	-0.049	-0.136	-0.064	-0.132	-0.172	-0.317	0.096
	NAb	-0.073	0.157	0.045	-0.041	0.117	-0.027	-0.119	-0.040	-0.043	-0.015	0.002	-0.082		-0.226	0.182	-0.117	-0.087	-0.124	-0.046	0.024	0.040	-0.021	0.055	0.208	0.104
	VL	0.084	0.057	0.024	0.153	-0.207	0.093	0.080	0.032	0.066	0.121	-0.259	0.184	-0.226		-0.070	0.223	0.087	0.373	0.120	0.153	-0.014	0.231	-0.326	-0.410	-0.042
	IL-8	-0.115	0.123	0.026	-0.177	-0.324	0.049	0.101	0.085	0.125	0.132	-0.111	-0.033	0.182	-0.070		-0.020	0.309	0.214	0.041	0.133	0.074	0.119	-0.159	-0.033	0.061
	H3	0.097	-0.142	0.351	0.047	0.260	-0.239	-0.260	0.262	-0.219	0.083	-0.003	-0.010	-0.117	0.223	-0.020		0.394	-0.037	0.530	0.303	0.170	0.233	-0.085	0.224	0.155
	cH3	0.151	0.184	-0.039	-0.023	-0.017	0.057	0.000	-0.064	0.071	0.176	-0.205	-0.164	-0.087	0.087	0.309	0.394		0.216	0.064	0.195	0.042	0.183	-0.068	-0.117	0.116
	MPO	-0.018	0.065	-0.021	0.039	-0.290	-0.145	-0.039	0.374	-0.017	0.481	-0.551	0.090	-0.124	0.373	0.214	-0.037	0.216		0.002	0.165	0.050	0.190	-0.220	-0.386	0.320
	hNE	0.050	-0.290	0.453	-0.002	0.318	-0.307	-0.298	0.211	-0.226	0.015	-0.068	-0.049	-0.046	0.120	0.041	0.530	0.064	0.002		0.100	0.014	0.129	-0.064	0.372	0.324
	FLCκ	0.207	-0.058	0.232	-0.039	0.174	-0.296	-0.427	0.239	-0.284	0.092	-0.196	-0.136	0.024	0.153	0.133	0.303	0.195	0.165	0.100		0.748	0.582	-0.164	0.067	0.424
	FLCλ	0.198	-0.089	0.252	-0.056	0.185	-0.322	-0.430	0.107	-0.256	0.011	-0.110	-0.064	0.040	-0.014	0.074	0.170	0.042	0.050	0.014	0.748		-0.031	-0.138	0.004	0.322
	FLCκ/FLCλ	0.093	0.036	0.045	-0.045	0.001	-0.119	-0.191	0.186	-0.114	0.068	-0.098	-0.132	-0.021	0.231	0.119	0.233	0.183	0.190	0.129	0.582	-0.031		-0.137	0.055	0.226
	MAP(min)	-0.106	-0.229	-0.047	-0.102	0.095	-0.081	-0.021	-0.081	0.138	-0.051	0.186	-0.172	0.055	-0.326	-0.159	-0.085	-0.068	-0.220	-0.064	-0.164	-0.138	-0.137		0.184	-0.113
	PUUV-N-IgG	-0.085	-0.060	0.175	-0.030	0.241	-0.036	-0.033	0.097	0.025	0.073	0.116	-0.317	0.208	-0.410	-0.033	0.224	-0.117	-0.386	0.372	0.067	0.004	0.055	0.184		-0.075
	Sev(scale)	0.106	-0.076	0.283	-0.106	0.062	-0.577	-0.652	0.447	-0.362	0.127	-0.375	0.096	0.104	-0.042	0.061	0.155	0.116	0.320	0.324	0.424	0.322	0.226	-0.113	-0.075	
**Scaled dataset**	Age		-0.211	0.322	-0.058	0.141	-0.337	-0.455	-0.028	-0.323	-0.197	0.333	-0.095	-0.133	0.061	-0.134	0.077	0.088	-0.078	-0.039	0.243	0.254	0.100	-0.003	-0.167	0.088
	sex	-0.211		-0.173	-0.015	-0.032	0.326	0.298	-0.276	-0.161	-0.420	0.075	-0.170	0.174	0.062	0.160	-0.192	0.123	-0.018	-0.151	-0.021	-0.038	0.032	-0.198	0.125	-0.156
	D(t)	0.322	-0.173		0.257	0.482	-0.739	-0.624	0.403	-0.667	0.006	-0.128	-0.066	0.138	-0.052	-0.036	0.164	-0.073	0.081	0.328	0.258	0.264	0.109	-0.072	0.088	0.439
	D(f)	-0.058	-0.015	0.257		0.173	-0.027	0.153	0.000	-0.087	-0.047	0.115	0.185	-0.237	0.103	-0.223	0.088	-0.034	0.003	-0.024	-0.038	-0.079	0.001	-0.264	-0.085	-0.259
	FDBH	0.141	-0.032	0.482	0.173		-0.376	-0.466	0.136	-0.332	-0.227	0.132	-0.253	0.026	-0.179	-0.322	0.119	-0.012	-0.173	0.162	0.198	0.257	-0.038	0.129	0.157	0.322
	eGFR(min)	-0.337	0.326	-0.739	-0.027	-0.376		0.854	-0.443	0.503	-0.084	0.022	0.185	-0.154	0.090	0.106	-0.306	0.053	-0.184	-0.319	-0.260	-0.286	-0.135	-0.231	-0.098	-0.519
	eGFR	-0.455	0.298	-0.624	0.153	-0.466	0.854		-0.325	0.519	0.015	0.047	0.320	-0.120	0.072	0.144	-0.365	-0.006	-0.070	-0.242	-0.391	-0.401	-0.168	-0.203	-0.040	-0.659
	WBC(max)	-0.028	-0.276	0.403	0.000	0.136	-0.443	-0.325		-0.211	0.505	-0.374	0.011	0.186	0.071	0.104	0.259	-0.049	0.401	0.301	0.184	0.034	0.176	-0.037	0.170	0.446
	HCT(min)	-0.323	-0.161	-0.667	-0.087	-0.332	0.503	0.519	-0.211		0.248	0.098	0.081	-0.212	0.117	0.152	-0.186	0.066	-0.014	-0.153	-0.381	-0.341	-0.180	0.123	-0.103	-0.355
	HCT(max)	-0.197	-0.420	0.006	-0.047	-0.227	-0.084	0.015	0.505	0.248		-0.496	0.125	-0.066	0.143	0.152	0.247	0.147	0.507	0.339	0.035	-0.060	0.057	-0.015	-0.060	0.287
	TC(min)	0.333	0.075	-0.128	0.115	0.132	0.022	0.047	-0.374	0.098	-0.496		-0.061	0.044	-0.238	-0.109	-0.237	-0.168	-0.559	-0.464	-0.194	-0.092	-0.138	0.176	-0.024	-0.487
	CRP(max)	-0.095	-0.170	-0.066	0.185	-0.253	0.185	0.320	0.011	0.081	0.125	-0.061		-0.168	0.130	-0.039	-0.093	-0.209	0.101	0.075	-0.149	-0.093	-0.098	-0.360	-0.222	-0.170
	NAb	-0.133	0.174	0.138	-0.237	0.026	-0.154	-0.120	0.186	-0.212	-0.066	0.044	-0.168		-0.267	0.197	-0.284	-0.106	-0.128	0.152	0.037	0.072	-0.014	0.238	0.438	0.233
	VL	0.061	0.062	-0.052	0.103	-0.179	0.090	0.072	0.071	0.117	0.143	-0.238	0.130	-0.267		-0.110	0.224	0.078	0.371	0.099	0.180	-0.029	0.299	-0.345	-0.411	-0.053
	IL-8	-0.134	0.160	-0.036	-0.223	-0.322	0.106	0.144	0.104	0.152	0.152	-0.109	-0.039	0.197	-0.110		-0.056	0.290	0.208	-0.047	0.128	0.068	0.112	-0.105	-0.037	0.008
	H3	0.077	-0.192	0.164	0.088	0.119	-0.306	-0.365	0.259	-0.186	0.247	-0.237	-0.093	-0.284	0.224	-0.056		0.380	0.345	0.123	0.316	0.178	0.237	-0.058	-0.290	0.245
	cH3	0.088	0.123	-0.073	-0.034	-0.012	0.053	-0.006	-0.049	0.066	0.147	-0.168	-0.209	-0.106	0.078	0.290	0.380		0.198	0.077	0.214	0.071	0.169	-0.069	-0.077	0.057
	MPO	-0.078	-0.018	0.081	0.003	-0.173	-0.184	-0.070	0.401	-0.014	0.507	-0.559	0.101	-0.128	0.371	0.208	0.345	0.198		0.372	0.129	-0.011	0.222	-0.227	-0.280	0.236
	hNE	-0.039	-0.151	0.328	-0.024	0.162	-0.319	-0.242	0.301	-0.153	0.339	-0.464	0.075	0.152	0.099	-0.047	0.123	0.077	0.372		0.110	0.014	0.128	-0.024	0.151	0.451
	FLCκ	0.243	-0.021	0.258	-0.038	0.198	-0.260	-0.391	0.184	-0.381	0.035	-0.194	-0.149	0.037	0.180	0.128	0.316	0.214	0.129	0.110		0.725	0.627	-0.115	0.029	0.407
	FLCλ	0.254	-0.038	0.264	-0.079	0.257	-0.286	-0.401	0.034	-0.341	-0.060	-0.092	-0.093	0.072	-0.029	0.068	0.178	0.071	-0.011	0.014	0.725		-0.010	-0.091	-0.031	0.304
	FLCκ/FLCλ	0.100	0.032	0.109	0.001	-0.038	-0.135	-0.168	0.176	-0.180	0.057	-0.138	-0.098	-0.014	0.299	0.112	0.237	0.169	0.222	0.128	0.627	-0.010		-0.088	0.049	0.227
	MAP(min)	-0.003	-0.198	-0.072	-0.264	0.129	-0.231	-0.203	-0.037	0.123	-0.015	0.176	-0.360	0.238	-0.345	-0.105	-0.058	-0.069	-0.227	-0.024	-0.115	-0.091	-0.088		0.371	0.035
	PUUV-N-IgG	-0.167	0.125	0.088	-0.085	0.157	-0.098	-0.040	0.170	-0.103	-0.060	-0.024	-0.222	0.438	-0.411	-0.037	-0.290	-0.077	-0.280	0.151	0.029	-0.031	0.049	0.371		0.223
	Sev(scale)	0.088	-0.156	0.439	-0.259	0.322	-0.519	-0.659	0.446	-0.355	0.287	-0.487	-0.170	0.233	-0.053	0.008	0.245	0.057	0.236	0.451	0.407	0.304	0.227	0.035	0.223	

Table S3. p-Values for the correlation for all variables for both the basic and scaled data. For abbreviations, see Supplementary Table S1.

	Variable	Age	sex	D(t)	D(f)	FDBH	eGFR(min)	eGFR	WBC(max)	HCT(min)	HCT(max)	TC(min)	CRP(max)	NAb	VL	IL-8	H3	cH3	MPO	hNE	FLCκ	FLCλ	FLCκ/FLCλ	MAP(min)	PUUV-N-IgG	Sev(scale)
**Basic dataset**	Age		0.841	**0.000**	0.311	0.079	**0.001**	**0.000**	0.832	**0.000**	**0.023**	0.088	0.060	0.437	0.543	0.415	0.353	0.285	0.873	0.655	0.129	0.146	0.500	0.258	0.401	0.257
	sex	0.841		0.385	0.875	0.397	**0.034**	0.233	**0.028**	**0.006**	**0.026**	0.154	0.795	0.092	0.677	0.386	0.174	0.191	0.556	**0.008**	0.672	0.519	0.793	**0.014**	0.555	0.416
	D(t)	**0.000**	0.385		**0.000**	**0.000**	**0.000**	**0.000**	**0.000**	**0.000**	0.795	0.626	0.501	0.634	0.864	0.854	**0.001**	0.781	0.851	**0.000**	0.088	0.063	0.743	0.617	0.084	**0.002**
	D(f)	0.311	0.875	**0.000**		**0.004**	0.784	0.581	0.846	0.161	0.942	0.888	**0.004**	0.667	0.264	0.210	0.658	0.872	0.723	0.985	0.776	0.685	0.745	0.281	0.771	0.262
	FDBH	0.079	0.397	**0.000**	**0.004**		**0.004**	**0.000**	0.840	0.241	**0.041**	**0.002**	**0.005**	0.216	0.129	**0.019**	**0.012**	0.905	**0.007**	**0.004**	0.204	0.176	0.995	0.319	**0.017**	0.515
	eGFR(min)	**0.001**	**0.034**	**0.000**	0.784	**0.004**		**0.000**	**0.000**	**0.000**	0.788	0.766	0.741	0.774	0.500	0.728	**0.021**	0.687	0.186	**0.005**	**0.028**	**0.016**	0.388	0.392	0.723	**0.000**
	eGFR	**0.000**	0.233	**0.000**	0.581	**0.000**	**0.000**		**0.000**	**0.000**	0.329	0.795	0.270	0.217	0.569	0.484	**0.014**	1.000	0.732	**0.008**	**0.001**	**0.001**	0.170	0.828	0.754	**0.000**
	WBC(max)	0.832	**0.028**	**0.000**	0.846	0.840	**0.000**	**0.000**		**0.016**	**0.000**	**0.000**	0.732	0.673	0.816	0.554	**0.011**	0.650	**0.000**	0.058	0.082	0.439	0.178	0.393	0.340	**0.000**
	HCT(min)	**0.000**	**0.006**	**0.000**	0.161	0.241	**0.000**	**0.000**	**0.016**		**0.005**	0.058	**0.007**	0.645	0.636	0.380	**0.035**	0.617	0.879	**0.041**	**0.037**	0.062	0.411	0.142	0.809	**0.000**
	HCT(max)	**0.023**	**0.026**	0.795	0.942	**0.041**	0.788	0.329	**0.000**	**0.005**		**0.000**	0.383	0.870	0.384	0.355	0.427	0.212	**0.000**	0.895	0.506	0.937	0.628	0.591	0.477	0.178
	TC(min)	0.088	0.154	0.626	0.888	**0.002**	0.766	0.795	**0.000**	0.058	**0.000**		**0.031**	0.982	0.059	0.439	0.976	0.144	**0.000**	0.549	0.156	0.427	0.482	**0.049**	0.258	**0.000**
	CRP(max)	0.060	0.795	0.501	**0.004**	**0.005**	0.741	0.270	0.732	**0.007**	0.383	**0.031**		0.382	0.183	0.816	0.921	0.245	0.413	0.663	0.326	0.644	0.341	0.067	**0.001**	0.308
	NAb	0.437	0.092	0.634	0.667	0.216	0.774	0.217	0.673	0.645	0.870	0.982	0.382		0.097	0.197	0.264	0.539	0.259	0.681	0.861	0.770	0.881	0.560	**0.039**	0.265
	VL	0.543	0.677	0.864	0.264	0.129	0.500	0.569	0.816	0.636	0.384	0.059	0.183	0.097		0.621	0.112	0.538	**0.006**	0.391	0.264	0.917	0.089	**0.015**	**0.002**	0.763
	IL-8	0.415	0.386	0.854	0.210	**0.019**	0.728	0.484	0.554	0.380	0.355	0.439	0.816	0.197	0.621		0.889	**0.027**	0.128	0.772	0.347	0.602	0.400	0.261	0.816	0.667
	H3	0.353	0.174	**0.001**	0.658	**0.012**	**0.021**	**0.014**	**0.011**	**0.035**	0.427	0.976	0.921	0.264	0.112	0.889		**0.004**	0.739	**0.000**	**0.029**	0.229	0.096	0.422	**0.039**	0.138
	cH3	0.285	0.191	0.781	0.872	0.905	0.687	1.000	0.650	0.617	0.212	0.144	0.245	0.539	0.538	**0.027**	**0.004**		0.124	0.652	0.166	0.767	0.195	0.634	0.407	0.413
	MPO	0.873	0.556	0.851	0.723	**0.007**	0.186	0.732	**0.000**	0.879	**0.000**	**0.000**	0.413	0.259	**0.006**	0.128	0.739	0.124		0.988	0.237	0.720	0.173	**0.045**	**0.000**	**0.003**
	hNE	0.655	**0.008**	**0.000**	0.985	**0.004**	**0.005**	**0.008**	0.058	**0.041**	0.895	0.549	0.663	0.681	0.391	0.772	**0.000**	0.652	0.988		0.476	0.923	0.356	0.566	**0.001**	**0.003**
	FLCκ	0.129	0.672	0.088	0.776	0.204	**0.028**	**0.001**	0.082	**0.037**	0.506	0.156	0.326	0.861	0.264	0.347	**0.029**	0.166	0.237	0.476		**0.000**	**0.000**	0.233	0.629	**0.001**
	FLCλ	0.146	0.519	0.063	0.685	0.176	**0.016**	**0.001**	0.439	0.062	0.937	0.427	0.644	0.770	0.917	0.602	0.229	0.767	0.720	0.923	**0.000**		0.820	0.314	0.975	**0.016**
	FLCκ/FLCλ	0.500	0.793	0.743	0.745	0.995	0.388	0.170	0.178	0.411	0.628	0.482	0.341	0.881	0.089	0.400	0.096	0.195	0.173	0.356	**0.000**	0.820		0.317	0.691	0.097
	MAP(min)	0.258	**0.014**	0.617	0.281	0.319	0.392	0.828	0.393	0.142	0.591	**0.049**	0.067	0.560	**0.015**	0.261	0.422	0.634	**0.045**	0.566	0.233	0.314	0.317		0.068	0.230
	PUUV-N-IgG	0.401	0.555	0.084	0.771	**0.017**	0.723	0.754	0.340	0.809	0.477	0.258	**0.001**	**0.039**	**0.002**	0.816	**0.039**	0.407	**0.000**	**0.001**	0.629	0.975	0.691	0.068		0.459
	Sev(scale)	0.257	0.416	**0.002**	0.262	0.515	**0.000**	**0.000**	**0.000**	**0.000**	0.178	**0.000**	0.308	0.265	0.763	0.667	0.138	0.413	**0.003**	**0.003**	**0.001**	**0.016**	0.097	0.230	0.459	
**Scaled dataset**	Age		0.145	**0.024**	0.693	0.335	**0.018**	**0.001**	0.851	**0.024**	0.176	**0.020**	0.516	0.364	0.677	0.358	0.600	0.547	0.593	0.790	0.093	0.079	0.495	0.985	0.251	0.546
	sex	0.145		0.234	0.919	0.828	**0.022**	**0.037**	0.055	0.269	**0.003**	0.609	0.242	0.231	0.673	0.272	0.186	0.399	0.904	0.300	0.888	0.795	0.826	0.172	0.393	0.284
	D(t)	**0.024**	0.234		0.075	**0.000**	**0.000**	**0.000**	**0.004**	**0.000**	0.967	0.380	0.650	0.346	0.721	0.805	0.260	0.620	0.581	**0.021**	0.074	0.067	0.456	0.623	0.546	**0.002**
	D(f)	0.693	0.919	0.075		0.235	0.852	0.293	1.000	0.551	0.747	0.432	0.204	0.102	0.482	0.124	0.547	0.816	0.983	0.868	0.796	0.591	0.993	0.067	0.560	0.072
	FDBH	0.335	0.828	**0.000**	0.235		**0.008**	**0.001**	0.351	**0.020**	0.117	0.365	0.079	0.860	0.220	**0.024**	0.417	0.933	0.235	0.267	0.172	0.074	0.794	0.376	0.282	**0.024**
	eGFR(min)	**0.018**	**0.022**	**0.000**	0.852	**0.008**		**0.000**	**0.001**	**0.000**	0.568	0.881	0.204	0.292	0.536	0.469	**0.032**	0.717	0.206	**0.025**	0.071	**0.046**	0.356	0.110	0.503	**0.000**
	eGFR	**0.001**	**0.037**	**0.000**	0.293	**0.001**	**0.000**		**0.023**	**0.000**	0.921	0.746	**0.025**	0.410	0.624	0.323	**0.010**	0.966	0.634	0.094	**0.005**	**0.004**	0.249	0.162	0.784	**0.000**
	WBC(max)	0.851	0.055	**0.004**	1.000	0.351	**0.001**	**0.023**		0.146	**0.000**	**0.008**	0.941	0.200	0.627	0.477	0.072	0.737	**0.004**	**0.036**	0.204	0.816	0.226	0.802	0.243	**0.001**
	HCT(min)	**0.024**	0.269	**0.000**	0.551	**0.020**	**0.000**	**0.000**	0.146		0.085	0.502	0.579	0.144	0.424	0.298	0.201	0.651	0.923	0.295	**0.007**	**0.017**	0.217	0.400	0.483	**0.012**
	HCT(max)	0.176	**0.003**	0.967	0.747	0.117	0.568	0.921	**0.000**	0.085		**0.000**	0.393	0.652	0.327	0.296	0.088	0.315	**0.000**	**0.017**	0.814	0.681	0.700	0.919	0.680	**0.046**
	TC(min)	**0.020**	0.609	0.380	0.432	0.365	0.881	0.746	**0.008**	0.502	**0.000**		0.678	0.762	0.100	0.454	0.101	0.248	**0.000**	**0.001**	0.181	0.528	0.346	0.225	0.872	**0.000**
	CRP(max)	0.516	0.242	0.650	0.204	0.079	0.204	**0.025**	0.941	0.579	0.393	0.678		0.249	0.373	0.792	0.525	0.149	0.490	0.609	0.308	0.526	0.501	**0.011**	0.126	0.242
	NAb	0.364	0.231	0.346	0.102	0.860	0.292	0.410	0.200	0.144	0.652	0.762	0.249		0.063	0.174	**0.048**	0.468	0.380	0.298	0.801	0.625	0.923	0.100	**0.002**	0.108
	VL	0.677	0.673	0.721	0.482	0.220	0.536	0.624	0.627	0.424	0.327	0.100	0.373	0.063		0.451	0.122	0.592	**0.009**	0.498	0.215	0.842	**0.037**	**0.015**	**0.003**	0.719
	IL-8	0.358	0.272	0.805	0.124	**0.024**	0.469	0.323	0.477	0.298	0.296	0.454	0.792	0.174	0.451		0.700	**0.043**	0.152	0.750	0.381	0.644	0.443	0.474	0.798	0.956
	H3	0.600	0.186	0.260	0.547	0.417	**0.032**	**0.010**	0.072	0.201	0.088	0.101	0.525	**0.048**	0.122	0.700		**0.007**	**0.015**	0.401	**0.027**	0.222	0.101	0.691	**0.043**	0.090
	cH3	0.547	0.399	0.620	0.816	0.933	0.717	0.966	0.737	0.651	0.315	0.248	0.149	0.468	0.592	**0.043**	**0.007**		0.173	0.598	0.139	0.626	0.244	0.638	0.598	0.698
	MPO	0.593	0.904	0.581	0.983	0.235	0.206	0.634	**0.004**	0.923	**0.000**	**0.000**	0.490	0.380	**0.009**	0.152	**0.015**	0.173		**0.009**	0.377	0.942	0.126	0.116	0.052	0.103
	hNE	0.790	0.300	**0.021**	0.868	0.267	**0.025**	0.094	**0.036**	0.295	**0.017**	**0.001**	0.609	0.298	0.498	0.750	0.401	0.598	**0.009**		0.453	0.922	0.382	0.871	0.299	**0.001**
	FLCκ	0.093	0.888	0.074	0.796	0.172	0.071	**0.005**	0.204	**0.007**	0.814	0.181	0.308	0.801	0.215	0.381	**0.027**	0.139	0.377	0.453		**0.000**	**0.000**	0.430	0.842	**0.004**
	FLCλ	0.079	0.795	0.067	0.591	0.074	**0.046**	**0.004**	0.816	**0.017**	0.681	0.528	0.526	0.625	0.842	0.644	0.222	0.626	0.942	0.922	**0.000**		0.948	0.535	0.832	**0.034**
	FLCκ/FLCλ	0.495	0.826	0.456	0.993	0.794	0.356	0.249	0.226	0.217	0.700	0.346	0.501	0.923	**0.037**	0.443	0.101	0.244	0.126	0.382	**0.000**	0.948		0.547	0.737	0.116
	MAP(min)	0.985	0.172	0.623	0.067	0.376	0.110	0.162	0.802	0.400	0.919	0.225	**0.011**	0.100	**0.015**	0.474	0.691	0.638	0.116	0.871	0.430	0.535	0.547		**0.009**	0.810
	PUUV-N-IgG	0.251	0.393	0.546	0.560	0.282	0.503	0.784	0.243	0.483	0.680	0.872	0.126	**0.002**	**0.003**	0.798	**0.043**	0.598	0.052	0.299	0.842	0.832	0.737	**0.009**		0.123
	Sev(scale)	0.546	0.284	**0.002**	0.072	**0.024**	**0.000**	**0.000**	**0.001**	**0.012**	**0.046**	**0.000**	0.242	0.108	0.719	0.956	0.090	0.698	0.103	**0.001**	**0.004**	**0.034**	0.116	0.810	0.123	

Table S4. Kruskal-Wallis test and general liner modelling (GLM) – analysis. The Kruskal-Wallis test included 4 levels of PUUV-NAb1 categories as the dependent variable (NAb≤320 = 29, NAb = 640 = 38, NAb = 1280 = 28, NAb ≥ 2560 = 21). GLM analysis was performed separately to PUUV-N-IgG1, PUUV-VL1, eGFRmin and eGFR as dependent variables. Variables of predictive significance marked in bold font.

		Kruskal-Wallis test			General linear model (GLM) analyses																			
		NAb			VL					eGFR(min)					eGFR					PUUV-N-IgG				
	Variable	DF	Chisq	p	DF	MS[1]	F	p	Est	DF	MS[1]	F	p	Est	DF	MS[1]	F	p	Est	DF	MS[1]	F	p	Est
**Basic dataset**	Age	3	3,15	0,37	1	563,16	0,03	0,87	-0,27	1	13255,30	10,81	**0,00**	-0,85	1	17392,61	13,44	**0,00**	-1,00	1	1,33	1,99	0,16	-0,01
	sex	3	3,75	0,29	1	34518,30	1,67	0,20	51,14	1	4956,96	3,81	0,05	14,08	1	1191,49	0,82	0,37	7,15	1	0,03	0,05	0,83	-0,04
	D(t)	3	0,50	0,92	1	289,26	0,01	0,91	-0,95	1	42888,35	44,72	**0,00**	-7,17	1	27473,69	22,93	**0,00**	-5,87	1	0,02	0,03	0,87	0,00
	D(f)	3	0,51	0,92	1	45337,18	2,21	0,14	14,18	1	67,15	0,05	0,82	-0,36	1	2601,21	1,81	0,18	2,25	1	0,62	0,91	0,34	-0,04
	FDBH	3	5,59	0,13	1	10297,89	0,49	0,49	-10,23	1	10321,16	7,74	**0,01**	-5,86	1	21320,65	14,74	**0,00**	-8,79	1	2,48	3,67	0,06	0,10
	eGFR(min)	3	3,75	0,29	1	7686,76	0,36	0,55	0,32	1					1	106806,55	241,28	**0,00**	0,86	1	0,42	0,61	0,44	0,00
	eGFR	3	6,67	0,08	1	9079,82	0,41	0,52	0,36	1	99548,05	241,28	**0,00**	0,81	1					1	0,27	0,38	0,54	0,00
	WBC(max)	3	0,48	0,92	1	2701,26	0,12	0,73	-1,59	1	35639,11	35,67	**0,00**	-3,70	1	26618,44	22,20	**0,00**	-3,26	1	0,40	0,59	0,45	0,01
	HCT(min)	3	0,66	0,88	1	9349,24	0,43	0,51	-360,40	1	37397,55	38,03	**0,00**	458,73	1	40660,76	38,22	**0,00**	491,78	1	0,68	1,01	0,32	2,14
	HCT(max)	3	1,20	0,75	1	10460,35	0,49	0,49	-284,29	1	1558,62	1,21	0,27	-74,23	1	3,22	0,00	0,96	-3,52	1	0,36	0,53	0,47	1,18
	TC(min)	3	7,41	0,06	1	61347,78	2,99	0,09	-1,28	1	402,48	0,31	0,58	0,05	1	61,52	0,04	0,84	-0,02	1	0,70	1,03	0,31	0,00
	CRP(max)	3	7,10	0,07	1	2969,50	0,14	0,71	0,15	1	987,00	0,75	0,39	-0,05	1	227,80	0,16	0,69	0,03	1	2,88	4,42	**0,04**	0,00
	NAb				1	25601,13	1,23	0,27	-0,02	1	368,49	0,27	0,60	0,00	1	6781,17	4,86	**0,03**	-0,01	1	1,71	2,57	0,11	0,00
	VL	3	9,17	**0,03**	1					1	527,11	0,36	0,55	0,02	1	569,42	0,41	0,52	0,02	1	1,88	2,51	0,12	0,00
	IL-8	3	1,62	0,65	1	69140,17	3,29	0,08	-0,27	1	341,49	0,23	0,63	8,63	1	71,04	0,05	0,82	3,96	1	0,10	0,13	0,72	0,14
	H3	3	1,66	0,65	1	285,52	0,01	0,91	0,64	1	2764,34	2,10	0,15	-0,99	1	3071,05	2,08	0,15	-1,06	1	1,61	2,42	0,12	0,03
	cH3	3	8,15	**0,04**	1	5050,80	0,23	0,64	-0,12	1	2,63	0,00	0,97	0,00	1	86,53	0,06	0,80	0,02	1	0,07	0,09	0,76	0,00
	MPO	3	7,91	**0,05**	1	22628,83	1,05	0,31	0,30	1	7447,60	6,12	**0,02**	-0,15	1	3536,27	2,56	0,11	-0,11	1	5,53	8,48	**0,00**	0,00
	hNE	3	3,22	0,36	1	3430,81	0,16	0,69	-0,10	1	7024,49	5,19	**0,03**	-0,10	1	6705,45	4,60	**0,04**	-0,10	1	2,55	3,62	0,06	0,00
	FLCκ	3	2,03	0,57	1	65473,80	3,26	0,08	1,46	1	7521,71	5,69	**0,02**	-0,50	1	16151,62	15,17	**0,00**	-0,73	1	0,09	0,12	0,74	0,00
	FLCλ	3	0,58	0,90	1	24783,89	1,19	0,28	1,44	1	4989,96	3,64	0,06	-0,64	1	8103,20	6,61	**0,01**	-0,85	1	0,00	0,01	0,94	0,00
	FLCκ/FLCλ	3	1,60	0,66	1	115977,68	6,06	**0,02**	93,85	1	634,47	0,44	0,51	-6,94	1	4387,85	3,37	0,07	-18,40	1	0,02	0,02	0,88	0,04
	MAP(min)	3	1,24	0,74	1	18441,19	0,88	0,35	-1,79	1	95,92	0,07	0,79	-0,08	1	411,75	0,28	0,60	0,18	1	1,79	2,70	0,10	0,01
	PUUV-N-IgG	3	7,37	0,06	1	51265,57	2,51	0,12	-35,47	1	822,35	0,61	0,44	3,56	1	568,56	0,38	0,54	2,98	1				
	Sev(scale)	3	1,78	0,62	1	968,00	0,05	0,83	-2,48	1	44345,69	46,89	**0,00**	-10,92	1	71966,47	92,92	**0,00**	-14,92	1	0,46	0,68	0,41	-0,04
**Scaled dataset**	Age	3	6,17	0,10	1	0,00	0,10	0,75	-0,04	1	0,45	4,90	**0,03**	-0,40	1	0,82	10,74	**0,00**	-0,54	1	0,06	1,18	0,28	-0,15
	sex	3	2,35	0,50	1	0,07	1,55	0,22	0,08	1	0,47	5,15	**0,03**	0,20	1	0,36	4,14	**0,05**	0,17	1	0,07	1,30	0,26	0,08
	D(t)	3	0,88	0,83	1	0,01	0,24	0,63	-0,09	1	1,43	20,09	**0,00**	-1,01	1	0,84	11,11	**0,00**	-0,78	1	0,06	1,15	0,29	-0,21
	D(f)	3	4,41	0,22	1	0,06	1,33	0,25	0,20	1	0,07	0,73	0,40	0,22	1	0,76	9,85	**0,00**	0,70	1	0,00	0,00	1,00	0,00
	FDBH	3	2,51	0,47	1	0,01	0,32	0,58	-0,09	1	0,74	7,40	**0,01**	-0,62	1	1,11	12,08	**0,00**	-0,76	1	0,01	0,21	0,65	-0,08
	eGFR(min)	3	2,78	0,43	1	0,03	0,63	0,43	0,08	1					1	2,66	72,63	**0,00**	0,75	1	0,04	0,66	0,42	0,09
	eGFR	3	3,82	0,28	1	0,02	0,54	0,46	0,08	1	2,88	72,63	**0,00**	0,82	1					1	0,04	0,69	0,41	0,09
	WBC(max)	3	1,15	0,76	1	0,00	0,05	0,82	-0,03	1	0,97	12,02	**0,00**	-0,64	1	0,79	10,30	**0,00**	-0,57	1	0,00	0,01	0,92	0,02
	HCT(min)	3	2,29	0,51	1	0,01	0,19	0,67	-0,07	1	0,91	11,07	**0,00**	0,73	1	0,97	13,23	**0,00**	0,75	1	0,01	0,15	0,70	0,07
	HCT(max)	3	3,38	0,34	1	0,02	0,37	0,55	-0,10	1	0,27	2,79	0,10	-0,39	1	0,07	0,72	0,40	-0,20	1	0,02	0,35	0,56	-0,11
	TC(min)	3	6,36	0,10	1	0,13	2,94	0,09	-0,20	1	0,01	0,09	0,77	0,05	1	0,01	0,12	0,73	0,06	1	0,01	0,17	0,68	0,05
	CRP(max)	3	4,23	0,24	1	0,00	0,02	0,89	0,02	1	0,01	0,08	0,78	0,07	1	0,32	3,62	0,06	0,41	1	0,22	4,25	**0,04**	-0,34
	NAb				1	0,04	1,00	0,32	-0,16	1	0,16	1,65	0,21	-0,31	1	0,43	5,07	**0,03**	-0,51	1	0,26	5,25	**0,03**	0,40
	VL	3	7,01	0,07	1					1	0,06	0,63	0,43	0,17	1	0,05	0,54	0,46	0,15	1	0,10	1,90	0,17	-0,22
	IL-8	3	2,01	0,57	1	0,15	3,55	0,07	-0,27	1	0,05	0,51	0,48	0,16	1	0,01	0,15	0,70	0,08	1	0,01	0,11	0,75	0,05
	H3	3	4,62	0,20	1	0,00	0,00	0,99	0,00	1	0,09	0,85	0,36	-0,23	1	0,17	1,87	0,18	-0,32	1	0,12	2,26	0,14	-0,27
	cH3	3	7,12	0,07	1	0,01	0,25	0,62	-0,07	1	0,00	0,04	0,85	-0,04	1	0,00	0,00	0,99	0,00	1	0,00	0,00	0,98	0,00
	MPO	3	3,65	0,30	1	0,04	0,78	0,38	0,11	1	0,31	3,22	0,08	-0,32	1	0,11	1,20	0,28	-0,19	1	0,23	4,44	**0,04**	-0,28
	hNE	3	3,67	0,30	1	0,01	0,33	0,57	-0,09	1	0,33	3,46	0,07	-0,43	1	0,24	2,69	0,11	-0,36	1	0,01	0,09	0,76	-0,05
	FLCκ	3	3,57	0,31	1	0,15	3,44	0,07	0,18	1	0,30	3,16	0,08	-0,26	1	0,82	10,71	**0,00**	-0,43	1	0,00	0,01	0,91	-0,01
	FLCλ	3	2,75	0,43	1	0,05	1,10	0,30	0,14	1	0,18	1,80	0,19	-0,26	1	0,37	4,24	**0,05**	-0,37	1	0,01	0,11	0,75	-0,05
	FLCκ/FLCλ	3	1,19	0,75	1	0,25	6,27	**0,02**	0,32	1	0,02	0,22	0,64	-0,09	1	0,22	2,40	0,13	-0,29	1	0,00	0,03	0,86	-0,03
	MAP(min)	3	5,33	0,15	1	0,03	0,65	0,42	-0,12	1	0,09	0,89	0,35	-0,21	1	0,08	0,84	0,36	-0,20	1	0,32	6,49	**0,01**	0,40
	PUUV-N-IgG	3	10,74	**0,01**	1	0,08	1,90	0,17	-0,18	1	0,07	0,66	0,42	0,16	1	0,06	0,69	0,41	0,16	1				
	Sev(scale)	3	3,60	0,31	1	0,01	0,20	0,66	-0,06	1	0,83	9,89	**0,00**	-0,57	1	1,69	29,40	**0,00**	-0,81	1	0,00	0,02	0,90	-0,02

[1]MS=Mean square

Table S5. Mixed model-analysis. The NAb was divided into 4 categories NAb≤320 = 29, NAb = 640 = 38, NAb = 1280 = 28, NAb ≥ 2560 = 21. VL had 3 categories: VL < 10, 10 ≤ VL <80 and VL ≥ 80. The 3 categories of PUUV-N-IgG were < 0.15, 0.15 ≤ PUUV-N-IgG < 0.9 and PUUV-N-IgG ≥ 0.9. In the analyses, we utilized mixed models with eGFR, HCT, WBC, TC or CRP as dependent variables, and time, the categorized variable, and the interaction of time and the categorized variable serving as the independent variables. There were 54 patients' observations in the time series.

			Fit Statistics	time*NAb[4]		time[1]		NAb[1]	
	Variable	N nonmiss	AIC	F Value	Pr > F	F Value	Pr > F	F Value	Pr > F
**NAb**	eGFR	195	1659,6	0,86	0,565	1,37	0,254	1,80	0,158
	HCT	191	-609,5	0,59	0,802	48,35	**<.0001**	0,89	0,451
	WBC	191	886,8	0,70	0,705	5,28	**0,002**	0,55	0,649
	TC	191	1742,7	2,95	**0,003**	50,60	**<.0001**	2,58	0,064
	CRP	190	1679,2	0,72	0,691	25,31	**<.0001**	2,03	0,122
			Fit Statistics	time*VL[3]		time[1]		VL[2]	
		N nonmiss	AIC	F Value	Pr > F	F Value	Pr > F	F Value	Pr > F
**VL**	eGFR	195	1688,5	1,12	0,352	1,78	0,153	1,70	0,194
	HCT	191	-638,4	1,61	0,149	49,54	**<.0001**	1,08	0,348
	WBC	191	891,0	1,94	0,078	5,03	**0,003**	0,77	0,468
	TC	191	1796,2	0,81	0,562	44,97	**<.0001**	0,74	0,482
	CRP	190	1709,8	1,52	0,178	28,79	**<.0001**	0,08	0,920
			Fit Statistics	time*PUUV-N-IgG[3]		time[1]		PUUV-N-IgG[2]	
		N nonmiss	AIC	F Value	Pr > F	F Value	Pr > F	F Value	Pr > F
**PUUV-N-IgG**	eGFR	195	1695,4	0,27	0,952	1,89	0,134	0,38	0,686
	HCT	191	-629,5	0,36	0,903	41,03	**<.0001**	**0,04**	0,964
	WBC	191	900,7	0,08	0,998	3,97	**0,010**	1,04	0,362
	TC	191	1773,7	5,01	***0,000***	52,19	**<.0001**	0,69	0,505
	CRP	190	1713,4	0,31	0,933	25,64	**<.0001**	1,50	0,233

Number of all observations in all models 216.

[1]DF=3, [2]DF=2 , [3]DF=6, [4]DF=9.

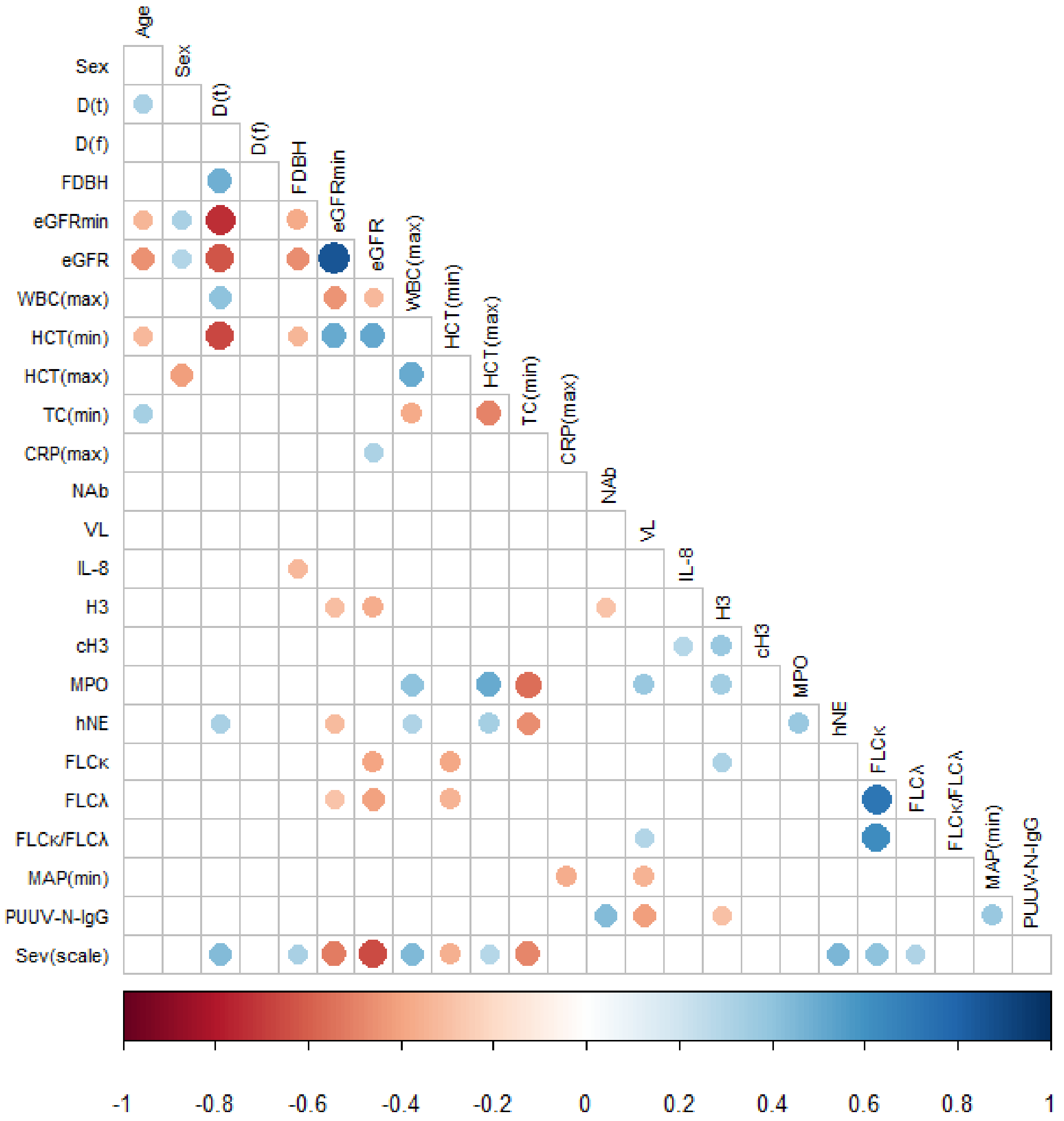

Figure S1. Spearman correlation plot of scaled dataset where missing data points in variables were excluded pairwise. Variable abbreviations are D(t) = Overall disease duration, D(F) = overall duration of fever, FDBH = Fever days before hopsitalization, eGFR = estimated glomerular filtration rate, WBC = white blood cell count, HCT = Hematocrit, TC = thrombocyte count, CRP = C-reactive protein, NAb = PUUV neutralizing antibodies, VL = PUUV viral load, IL-8 = Interleukin-8, H3 = Histone H3, cH3 = Citrullinated histone H3, MPO = Myeloperoxidase, hNE = Neutrophil elastase, FLCκ = Free light chain κ, FLCλ = Free light chain λ, MAP =mean arterial pressure, Sev(scale) = Severity scale The maximum or minimum values during the hospitalization are indicated as min or max, respectively and all other parameters are measured from 1st day of hospitalization. Only significant correlations at $p \leq 0.05$ are shown and the size of the dots are equivalent to the significance.

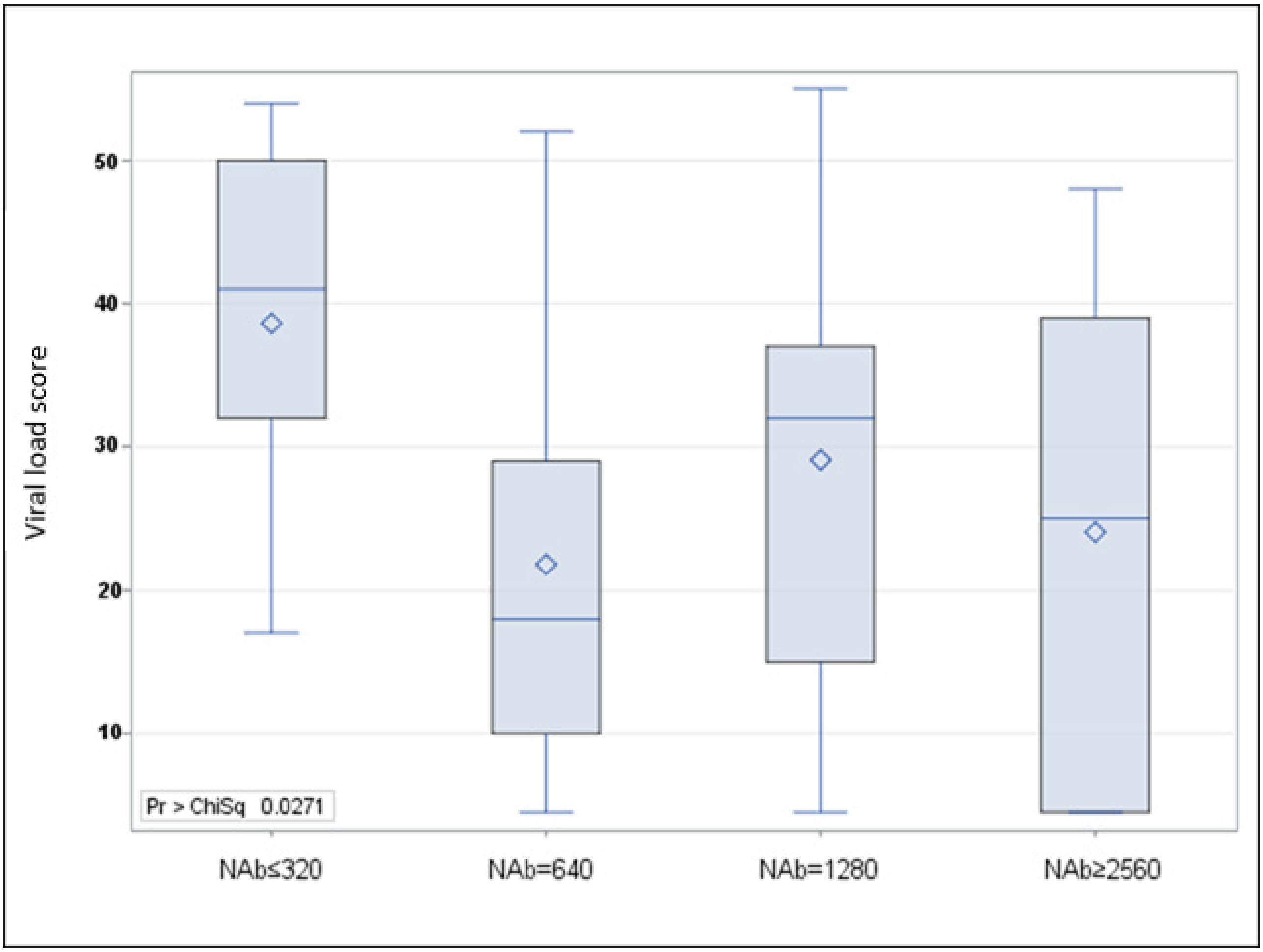

Figure S2. Distribution of Wilcoxon scores for VL in four groups of Nab. The group sizes are as follows: Nab ≤ 320 = 29, NAb = 640 = 38, NAb = 1280 = 28, NAb ≥ 2560 = 21

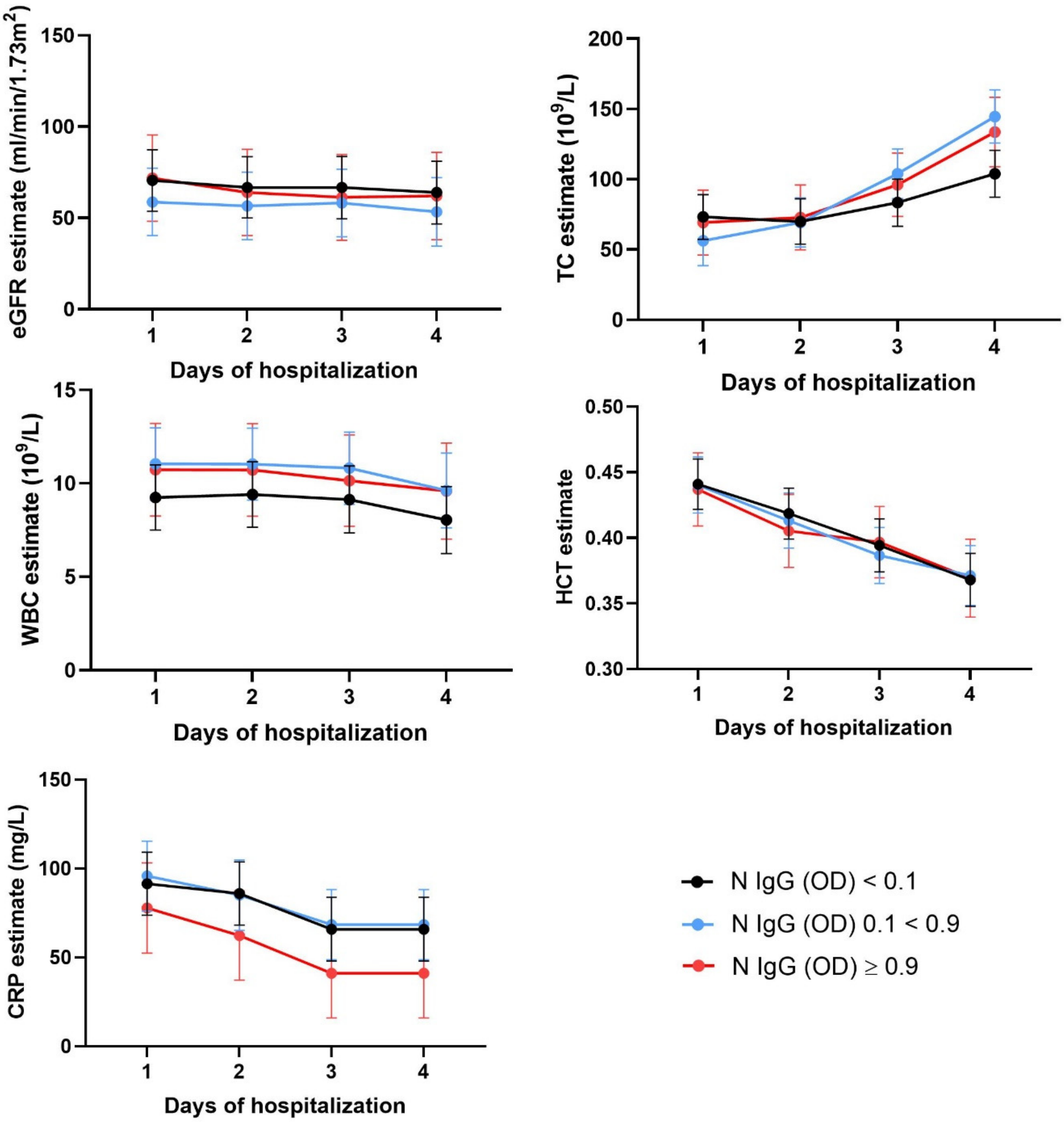

Figure S3. Time series graphs showing the impact of different categories of PUUV N-IgG at 1st day of hospitalization on eGFR, TC, WBC, HKR and CRP. The variables eGFR, TC, WBC, HCT and CRP were followed over time (the first 4 days of hopsitalization) and reported as mean estimates from the mixed model analysis. The error bars indicate upper and lower limits of the estimates.

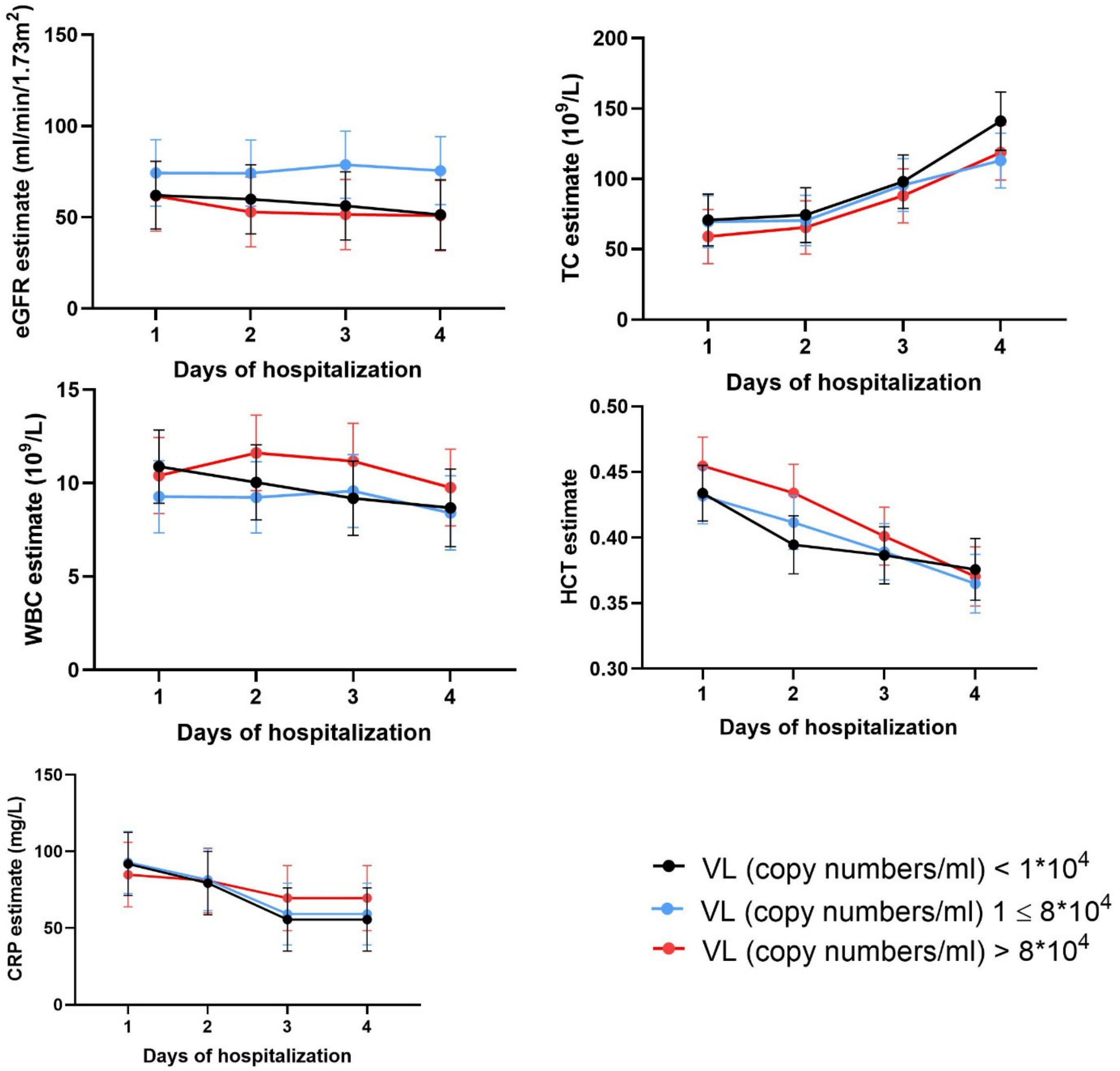

Figure S4. Time series graphs showing the impact of different categories of PUUV VL at 1st day of hospitalization on eGFR, TC, WBC, HCT and CRP. The variables eGFR, TC, WBC, HCT and CRP were followed over time (the first 4 days of hospitalization) and reported as mean estimates from the mixed model analysis. The error bars indicate upper and lower limits of the estimates.

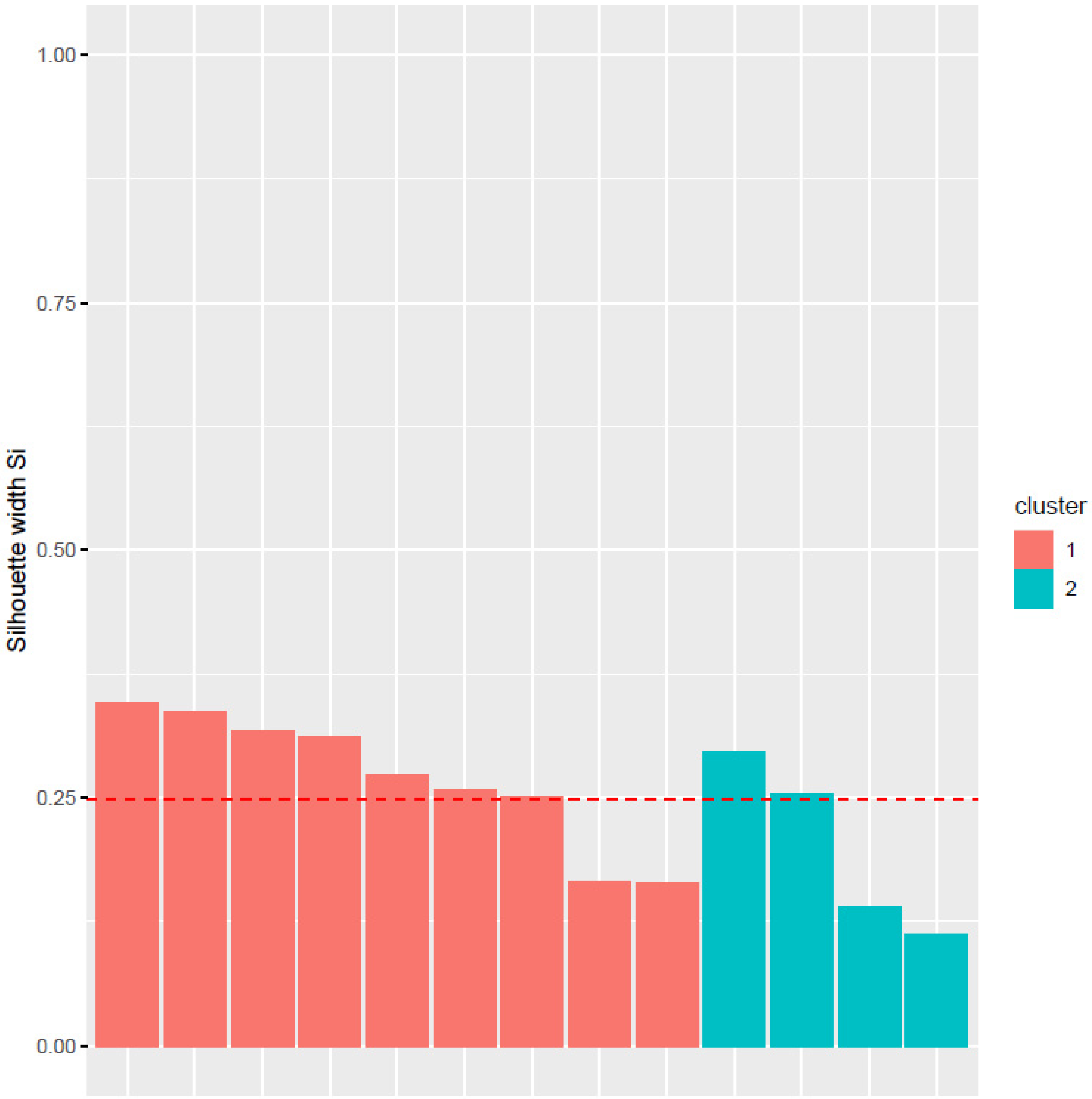

Figure S5. Silhouette plot of hierarchical cluster analysis. Average silhouette width 0.25. Cluster 1: 9 clusters, average silhouette width 0.27. Cluster 2: 4 clusters, average silhouette width 0.20. The clustering with the highest average silhouette width is considered to present the most appropriate clustering of the data points.
