# Supplementary figures and images for: Neutralizing Antibody Titers in Hospitalized Patients with Acute Puumala Orthohantavirus Infection Do Not Associate with Disease Severity
Source: Viruses. 2022 Apr 26;14(5):901. doi: 10.3390/v14050901 (PMC9143849; doi:10.3390/v14050901)

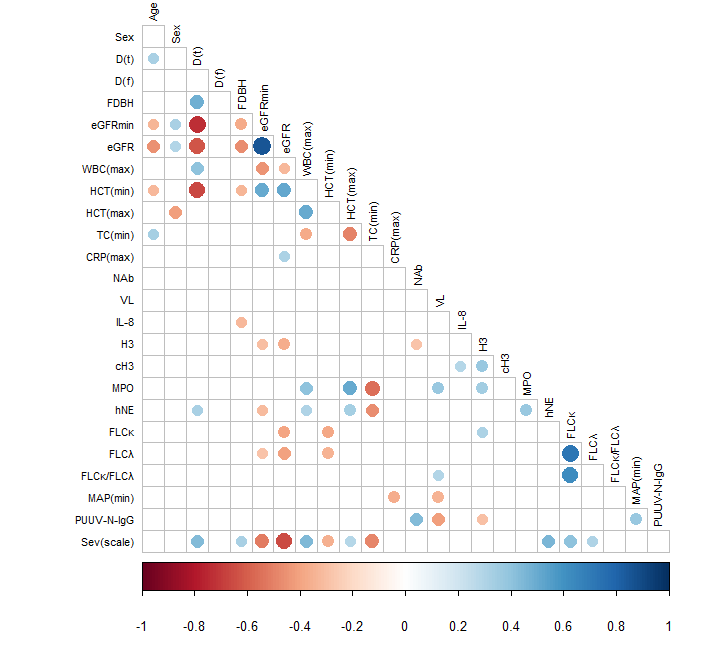

Supplement: Supplementary file 1 [file viruses-14-00901-s001.zip › Fig S1corrplot_scaled10032022.png]

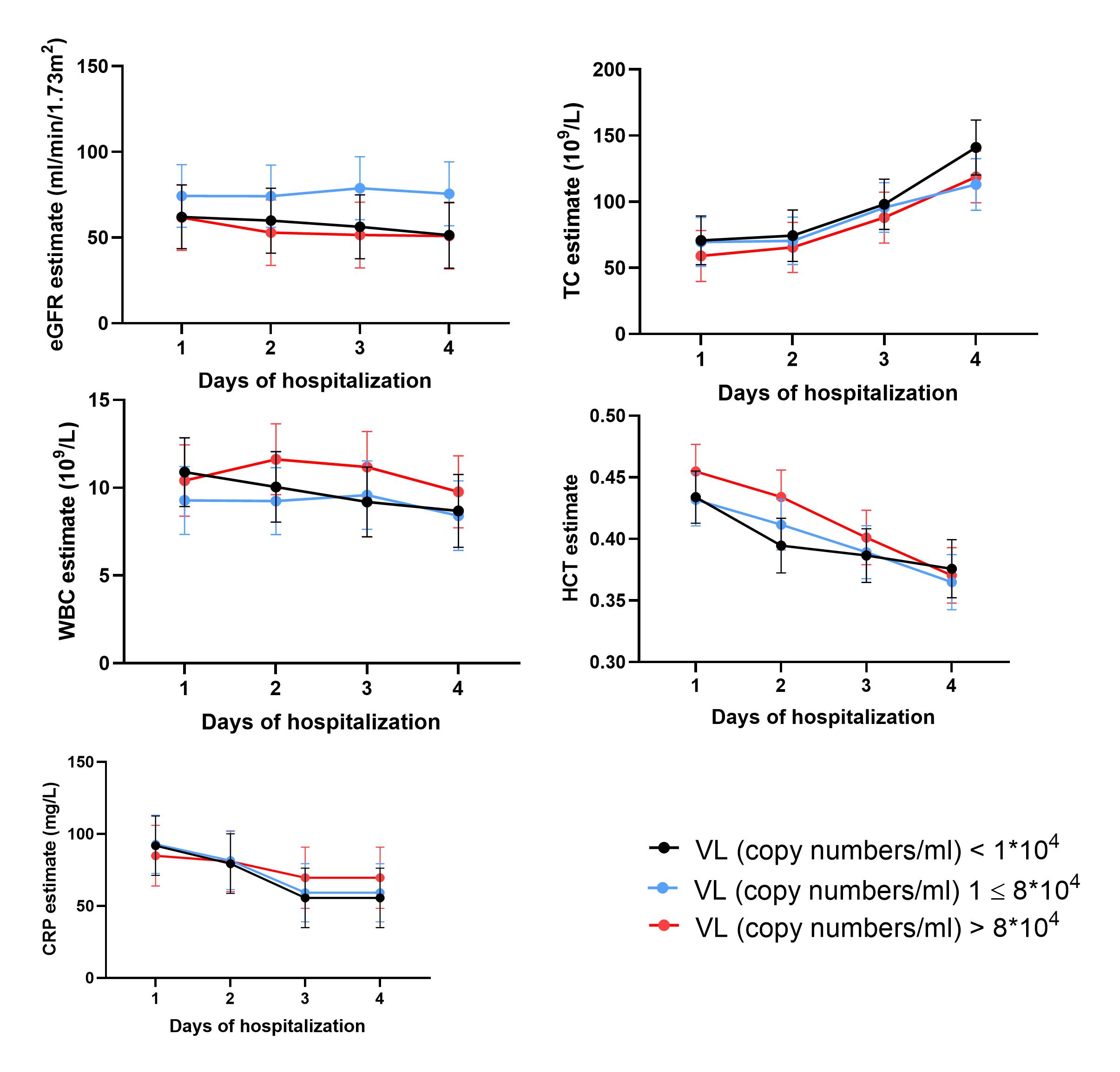

Supplement: Supplementary file 1 [file viruses-14-00901-s001.zip › fig S4.jpg]

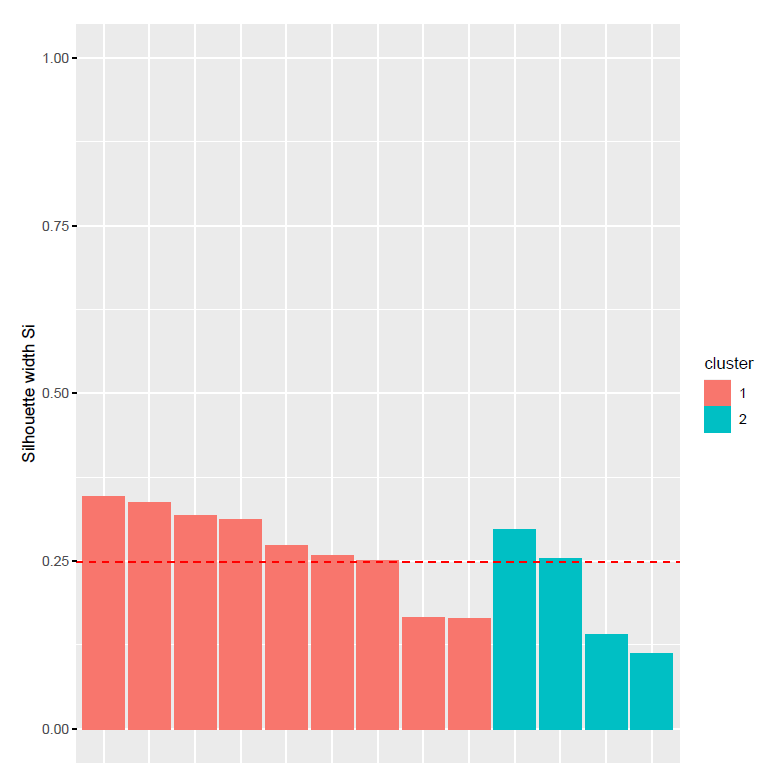

Supplement: Supplementary file 1 [file viruses-14-00901-s001.zip › Fig S5 Silhouette plot 15032022 (1).png]

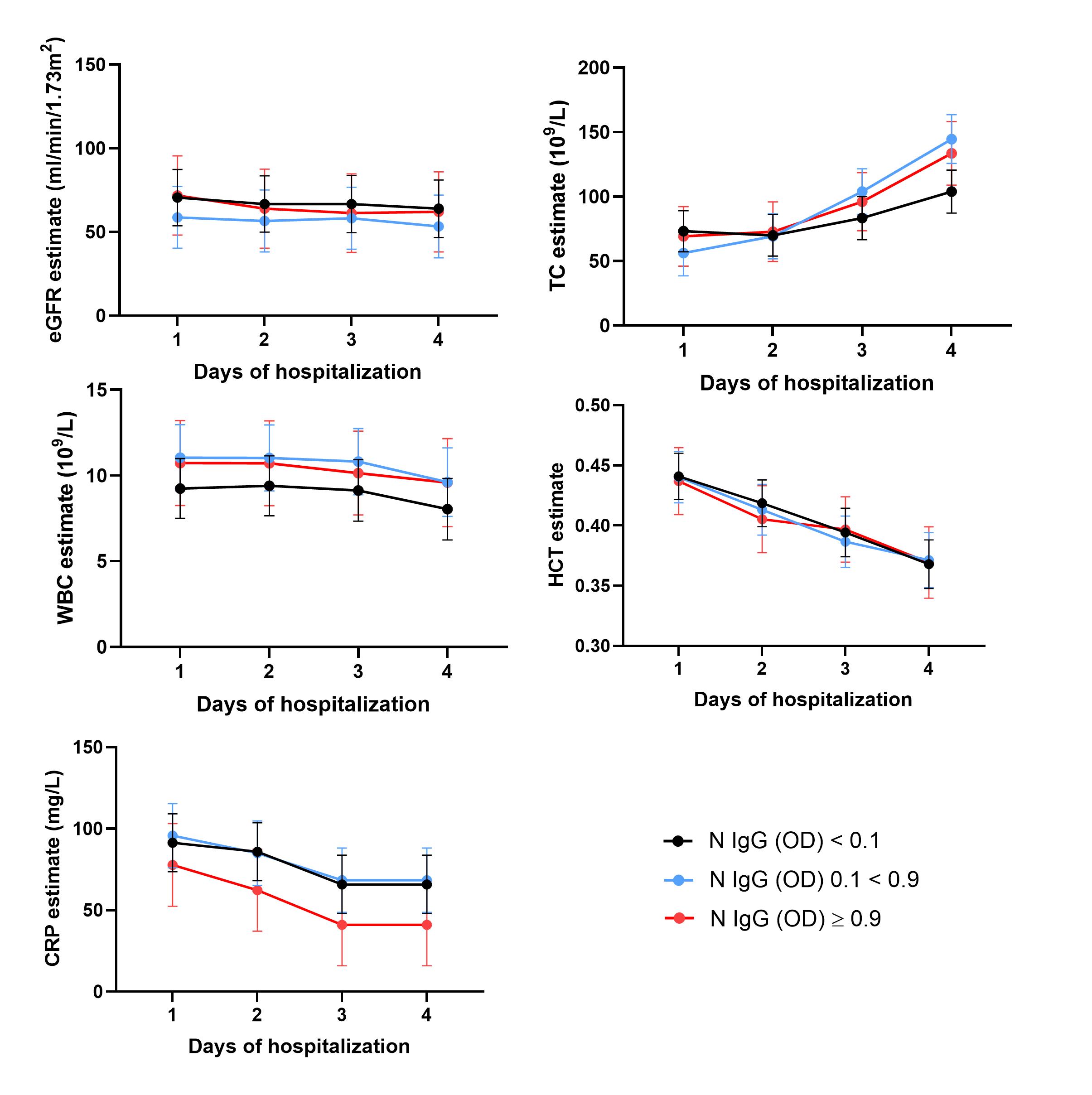

Supplement: Supplementary file 1 [file viruses-14-00901-s001.zip › Fig.S3.jpg]

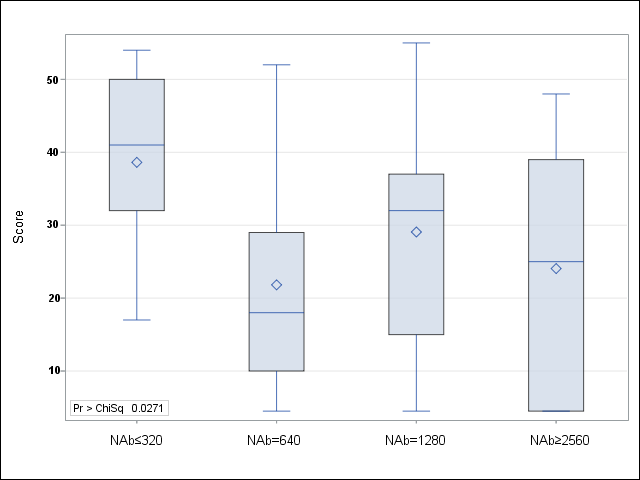

Supplement: Supplementary file 1 [file viruses-14-00901-s001.zip › FigS2WilcoxonBoxPlot15032022 (1).png]
